# Discovery and validation of information theory-based transcription factor and cofactor binding site motifs

**DOI:** 10.1093/nar/gkw1036

**Published:** 2016-11-28

**Authors:** Ruipeng Lu, Eliseos J. Mucaki, Peter K. Rogan

**Affiliations:** 1 Department of Computer Science, Western University, London, Ontario, N6A 5B7, Canada; 2 Department of Biochemistry, Western University, London, Ontario, N6A 5C1, Canada; 3 Department of Oncology, Western University, London, Ontario, N6A 4L6, Canada; 4 Cytognomix Inc., London, Ontario, N5X 3X5, Canada

## Abstract

Data from ChIP-seq experiments can derive the genome-wide binding specificities of transcription factors (TFs) and other regulatory proteins. We analyzed 765 ENCODE ChIP-seq peak datasets of 207 human TFs with a novel motif discovery pipeline based on recursive, thresholded entropy minimization. This approach, while obviating the need to compensate for skewed nucleotide composition, distinguishes true binding motifs from noise, quantifies the strengths of individual binding sites based on computed affinity and detects adjacent cofactor binding sites that coordinate with the targets of primary, immunoprecipitated TFs. We obtained contiguous and bipartite information theory-based position weight matrices (iPWMs) for 93 sequence-specific TFs, discovered 23 cofactor motifs for 127 TFs and revealed six high-confidence novel motifs. The reliability and accuracy of these iPWMs were determined via four independent validation methods, including the detection of experimentally proven binding sites, explanation of effects of characterized SNPs, comparison with previously published motifs and statistical analyses. We also predict previously unreported TF coregulatory interactions (e.g. TF complexes). These iPWMs constitute a powerful tool for predicting the effects of sequence variants in known binding sites, performing mutation analysis on regulatory SNPs and predicting previously unrecognized binding sites and target genes.

## INTRODUCTION

Transcription factors (TFs) interact with regulatory elements in genes to mediate positive or negative regulation of tissue- and stage-specific expression (1,2). TFs either directly bind to DNA by recognizing specific sequence motifs, or indirectly interact as partners (or cofactors) of sequence-specific TFs (3). Interactions between these two types of TFs, as well as between sequence-specific TFs, abound across the whole genome (3,4). For instance, NF-Y extensively coassociates with FOS over all chromatin states and CTCF extensively colocalizes with cohesins consisting of SMC1/SMC3 heterodimers and two non-SMC subunits RAD21 and SCC3 (5,6). The genome-wide distributions of both types of bound TFs have been analyzed by sequence analysis of immunoprecipitated chromatin (ChIP-seq) (7). ChIP-seq can identify the repertoire of binding site sequences in a genome, and often pull down binding sites of coregulatory cofactors.

Sequence-specific TFs either recognize contiguous sequence motifs, or form homodimeric or heterodimeric structures that contact half sites separated by gaps that together comprise bipartite binding sites (8). Although generally the binding sequences of TFs are well conserved, significant variability at most positions of their binding motifs characterizes most TFs. Information theory-based position weight matrices (iPWMs) can quantitatively and accurately describe these base preferences. A contiguous iPWM is derived from a set of aligned binding sites using Shannon information theory and a uniform background nucleotide composition (9,10). This approach may be more appropriate for defining binding sites than Relative Entropy because the contacts between the TF and the nucleotides do not depend on the background genomic composition (10,11). A bipartite iPWM consists of two contiguous, adjacent iPWMs, each corresponding to a half site, separated by a range of sequence gaps. The individual information content (*R_i_*) of a TF-bound sequence, which represents the affinity of the TF–DNA interaction, is the dot product between the binary matrix of the sequence and an iPWM of the TF (10). The *R*_sequence_ value of an iPWM is the mean of the *R_i_* values of all the binding site sequences used to compute the iPWM, and represents the average binding affinity (12). Our laboratory previously developed the Bipad software to generate bipartite (and contiguous) iPWMs from ChIP-seq data (8).

TF binding motifs have been derived from both experimental evidence and computational approaches. Weirauch *et al.* (13) measured TF binding by octanucleotide microarrays to infer sequence specificity from overlapping bound sequences for >1000 TFs encompassing 54 different DNA binding domain (DBD) classes. Jolma *et al.* (14) obtained 830 binding profiles representing 411 human and mouse TFs using high-throughput SELEX and ChIP sequencing. The oligonucleotide-based approach does not account for variable-length spacers in bipartite binding sites, and it may reconstruct potentially incorrect motifs that cannot be discriminated from correct binding site sequences. In addition, the set of octamers used in the DNA microarrays may not cover all possible binding site sequences (>8 nucleotides [nt]) recovered in the genome from ChIP-seq and there is no way to discover potential binding sites from TF cofactors. Wang *et al.* (3) carried out *de novo* motif discovery for 119 human TFs from 457 ChIP-seq datasets using the MEME-ChIP software suite, and Kheradpour *et al.* (15) provided a systematic motif analysis for 427 ChIP-seq datasets of 123 human TFs using five motif discovery tools. However, these studies did not generate bipartite motifs with half sites separated by gaps varying in length; more importantly, the derived motifs were only based upon strongest ChIP-seq signal peaks (top 500 or 250 peaks), effectively eliminating thousands of intermediate or weak binding events and biasing the resulting iPWMs toward high-affinity, consensus-like binding sites. This is necessary, as the sequences contained in the weakest ChIP-seq peaks may contribute low-complexity, likely non-functional sequences (i.e. noise) that can obfuscate the detection of true binding motifs. Extreme peak selection bias in the population of sites distorts the binding strengths estimated for individual sites (16).

We developed a motif discovery pipeline, Maskminent, by integrating recursive masking and thresholding the maximum number of ChIP-seq peaks into an entropy minimization framework. Bipad was modified to incorporate these features, and TF binding motifs were derived and validated for 765 ENCODE ChIP-seq datasets (1275 replicates) consisting of 207 human TFs. 93 primary and 23 cofactor binding motifs were successfully recovered and refined for 127 TFs. Reanalysis of the same data using the masking and thresholding techniques revealed many known and previously unreported TF cofactors; however, frequently our approach revealed cofactor motifs directly. These primary motifs were validated by comparing predicted with experimentally-detected true binding sites, explaining effects of characterized SNPs on binding site strengths, and through comparisons to an independent motif database.

## MATERIALS AND METHODS

### ENCODE ChIP-seq datasets

The ENCODE Consortium conducted ChIP-seq assays for human TFs and generated initial peak datasets for each replicate of each assay using a uniform peak calling pipeline (7,17). For some assays, these analyses produced optimal and conservative IDR-thresholded peaks after applying the IDR (irreproducible discovery rate) framework to the initial datasets to improve consistency of motifs obtained from multiple biological replicates. In addition, Factorbook (3,18) also reports motifs from refined datasets (limited to the top 500 peaks) generated by the SPP peak calling software (19).

We started with the IDR-thresholded peak datasets, because we found that these data are more likely to produce primary or cofactor motifs than the initial (i.e. unprocessed) datasets; they contain greater numbers of ChIP-seq peaks (and thus more binding sites) than the truncated SPP datasets. The initial, unfiltered datasets were examined if neither IDR-thresholded nor SPP datasets were available.

### The Maskminent motif discovery pipeline

Initially, iPWMs from ChIP-seq reads were derived by entropy minimization with Bipad (Supplementary Data). However, we noted that these iPWMs sometimes exhibited cofactor or noise motifs, rather than the expected primary motifs. In order to improve detection of primary motifs, the Maskminent software, which implements a generalization of the objective function used in Bipad, enables new motif discovery by recursively masking sequences detected by previous analyses of a ChIP-seq dataset while defining thresholds for inclusion of the maximum number of top peaks to eliminate peaks with lower signal intensities whose inclusion can result in emergence of noise over primary or cofactor motifs (Supplementary Data). Multiple ChIP-seq datasets from distinct cell lines for the same TF, if available, were examined for enriched sequence motifs to assess whether this approach was reproducible, and discover tissue-specific sequence preferences between these sources.

This masking technique, which contrasts with the likelihood approach used by MEME (20), provides a means of discovering additional conserved motifs adjacent to primary TF binding sites within the same datasets. The sequences detected by motifs found in previous iterations are masked and the next lowest entropy motif is derived. The coordinates of all the predicted binding sites in a dataset scanned with prior iPWMs are recorded and skipped in the subsequent reanalysis. The specified parameters include the length of the motif, number of Monte Carlo cycles used in entropy minimization, a motif masking file for recursion, and for bipartite binding sites, the lengths of the left and right motifs and the gap length range between the half sites (Supplementary Data). Once a motif is generated, another program, Scan, is used to detect binding sites in a DNA sequence and determine their respective information contents, or binding strengths.

**Figure 1.**
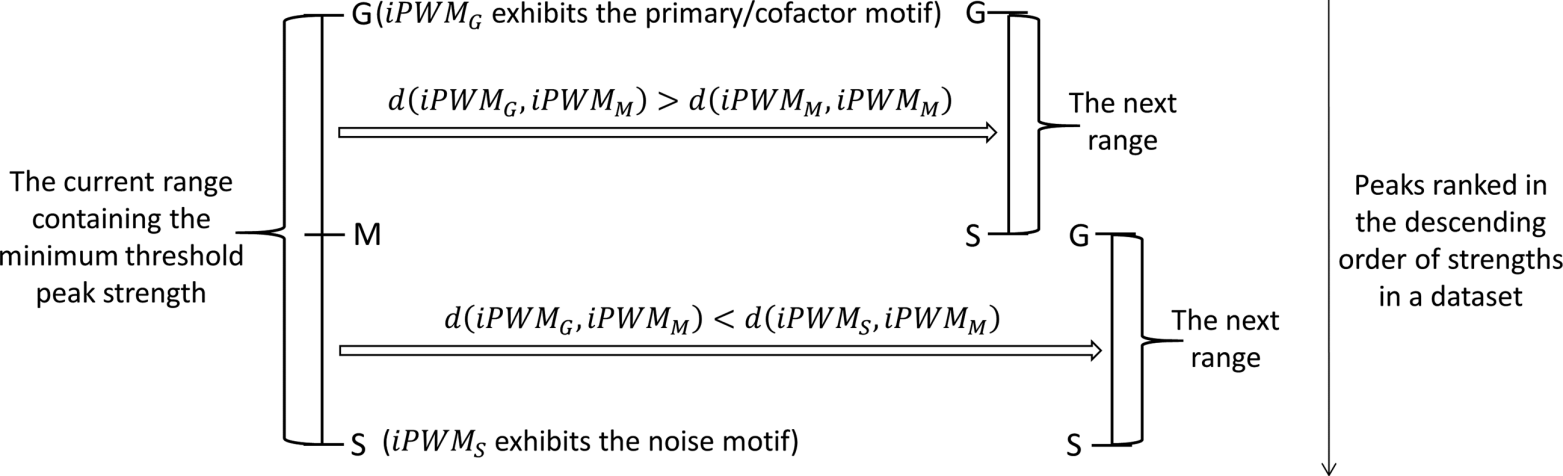
One iteration of the half-interval search used to refine the threshold peak strength. All peaks in the dataset are sorted in the descending order of signal strengths. $S$ is the smaller bound of the current range containing the minimum threshold that can generate the primary/cofactor motif, and $G$ is the greater bound (i.e. the current threshold). $G$ and $S$ are respectively initialized to the strength of the 200th peak and the strength of the last peak. $M$ is the strength of the peak at the mean (rounding to the nearest multiple of 500) of the number of top peaks above $G$ and the number of top peaks above $S$. $iPW{M_G}$, $iPW{M_S}$, $iPW{M_M}$ are respectively the iPWMs derived from the top peaks above $G$, $S$, $M$. $d( {iPW{M_G},iPW{M_M}} )$ is the Euclidean distance between $iPW{M_G}$ and $iPW{M_M}$, and $d( {iPW{M_S},iPW{M_M}} )$ is the Euclidean distance between $iPW{M_S}$ and $iPW{M_M}$. If $d( {iPW{M_G},iPW{M_M}} )$ is greater than $d( {iPW{M_S},iPW{M_M}} )$, $iPW{M_M}$ exhibits the noise motif and the minimum threshold is contained in the subrange from $G$ to $M$; if $d( {iPW{M_G},iPW{M_M}} )$ is smaller than $d( {iPW{M_S},iPW{M_M}} )$, $iPW{M_M}$ exhibits the primary/cofactor motif and the minimum threshold is contained in the subrange from $M$ to $S$. When the number of peaks contained in the range does not exceed 500, this half-interval search is stopped. The approximately minimum threshold that is returned is $G$ of the final range.

To eliminate noisy patterns that suppress the expected TF binding motifs due to ChIP-seq peaks with low signal strengths (i.e. read counts), the dataset is truncated based on signal strengths as follows (Figure 1). First, all the peaks are ranked in the descending order of strengths, and the top 200 peaks are selected. If the iPWM derived from the top 200 peaks exhibits the primary/cofactor motif, then the minimum threshold peak strength is contained within the range from the strength of the 200th peak (i.e. the initial value of $G$) to the peak with the weakest signal (i.e. the initial value of $S$). A half-interval search iterated over sets of progressively weaker peaks narrows this range until the number of peaks contained in the range is ≤500. The value of $G$ is the threshold peak signal strength above which the top peaks can still produce the primary/cofactor motif. The minimum threshold obtained for $G$ (i.e. the final value of $G$) defines the approximate peak set containing the maximum number of top peaks that can produce the primary/cofactor motif.

### Binding site motif validation

The methods used to evaluate the accuracy of our iPWMs include:

- To detect experimentally proven binding sites in known target genes, derived iPWMs were used to evaluate the *R_i_* value of each site;
- To predict changes in binding site strength, characterized variants were evaluated with the corresponding iPWMs. The predicted changes were compared with experimentally supported effects on TF binding or gene expression;
- The iPWMs were compared with the corresponding annotated motifs in the CIS-BP database (13) based on their normalized Euclidean distances;
- To distinguish true binding motifs from noise motifs, we delineated the relationship between *R_i_* values of binding sites discovered by the iPWM and their corresponding binding energy (i.e. higher *R_i_* values have lower binding energies) (Supplementary Data). Primary/cofactor motifs are expected to demonstrate this relationship, whereas noise motifs are not; that is, for primary/cofactor motifs, the linear regression fit between *R_i_* values and binding energy are expected to have slopes well below 0 which is the expected slope for noise motifs. After applying F-tests to evaluate this relationship, *F*-values for the two categories of motifs were compared using a Mann–Whitney U test.

## RESULTS

The derived iPWMs displayed primary motifs for 93 TFs (Supplementary Data), as well as 23 cofactor motifs for 127 primary TFs (Supplementary Data). We also describe 6 high-confidence novel motifs that have not been previously annotated in these ChIP-seq data (Supplementary Data).

The initial iPWMs directly exhibited primary motifs for 76 TFs and 18 cofactor motifs for 107 primary TFs. Thresholding the datasets revealed 31 primary motifs and 14 cofactors for 38 primary TFs. We used the masking technique to discover an additional 4 primary motifs; 7 cofactor motifs were also found in 21 datasets (Supplementary Data).

For each TF ChIP-seq dataset with a derived primary motif (*n* = 367), we determined the false positive detection rate from the null *R_i_* distribution, which is approximately Gaussian (12). The iPWM was used to scan for binding sites in a random 10 000 nucleotide sequence that conserved the mono- and dinucleotide composition as the dataset (Supplementary Data). The means of all null distributions range from −97.5 to −12.3 bits with standard deviations from 6.9 to 22.5 bits. The probabilities of observing a potentially functional binding site, i.e. with *R_i_* > 0, in these sequences range from 1.2E-7 to 0.06.

Similarly, the independence of contributions of each position in a binding site to the overall information content was analyzed for one iPWM of each primary motif. The total mutual information, which measures the interdependence between individual positions in the same binding site, was determined by summing the pairwise mutual information at each position (Supplementary Data). Then, the percentage of the total mutual information relative to the average information, *R*_sequence_, was determined. For 83 TFs (∼89.2%), <10% of the information present in the iPWM is dependent, and for 62 TFs (∼66.7%), <5% is dependent. Neglecting the interactions between positions introduces a minimal error into the calculation of *R_i_* values of binding sites, and would be expected to have little impact on assessment of the mutations in these sequences.

### Primary binding motifs

#### Contiguous iPWMs

**Figure 2.**
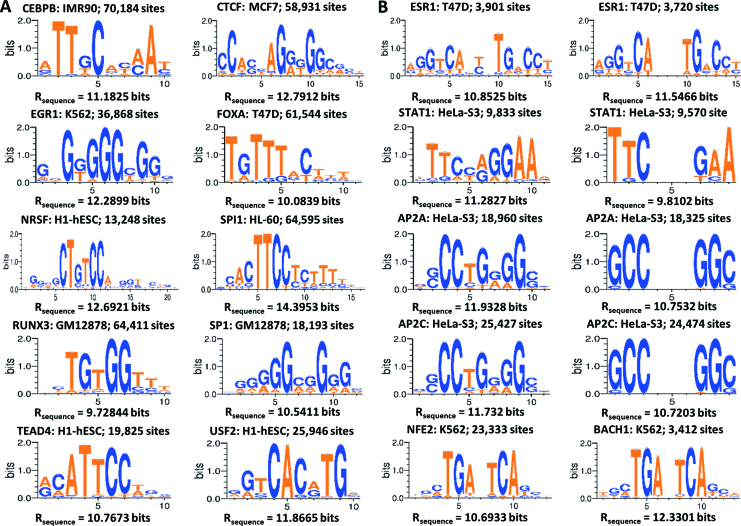
Sequence logos of contiguous (**A**) and bipartite (**B**) iPWMs. The TF name, and the cell line from which the iPWM was derived, and the number of binding sites that the iPWM is based upon are displayed. In (B), each of the first four rows includes a contiguous (left) iPWM and a bipartite (right) iPWM of one TF from the same dataset. The last row includes the bipartite iPWMs of NFE2 and BACH1. The bipartite search patterns, which are denoted by *l<a,b>r* (*l* and *r* are the lengths of the left and right half sites respectively, *a* and *b* are the minimum and maximum spacer lengths respectively), are 6<0,5>6, 3<2,4>3, 3<2,4>3, 3<2,4>3, 6<1,2>6 and 6<1,2>6 from top to bottom, respectively.

Correct iPWMs were successfully derived for 65 TFs with contiguous binding motifs, which are concordant with published descriptions of these motifs (3). All of these motifs can be characterized as degenerate and do not correspond to published consensus sequences. Consensus sequences miss TF binding sites of weak or intermediate strength (16). We determined the frequencies of such sequences appearing on a genome scale for 10 TFs by counting the peaks containing these sequences in their respective datasets (Figure 2A). Surprisingly, only 0.015–7.3% of all peaks contain binding sites with these sequences, demonstrating that these sites are extremely rare in ChIP-seq datasets. Thus, intermediate and low-affinity TF-DNA interactions are the most prevalent *in vivo* and are able to regulate gene expression (21).

#### Bipartite iPWMs

For 19 TFs, bipartite iPWMs were successfully derived and were in agreement with previously reported motifs. The following examples illustrate key insights that can be taken from bipartite modeling:

- El Marzouk *et al.* (22) demonstrated that ESR1 is able to recognize binding sites with half sites separated by nucleotide spacer lengths from 0 to 4 nt, in which sites containing a 3 nt spacer are most common and have the highest binding affinities. We allowed the spacer length to vary from 0 to 5 nt in bipartite iPWMs derived from the T47D cell line data. The resultant iPWMs show the documented predominant sequences and are palindromic. The bipartite iPWM exceeds the average information content of the corresponding contiguous iPWM prepared from the same dataset, and the dominant gap between half sites is 3 nt (Figure 2B). Nevertheless, 333 binding sites (∼9%) in this iPWM exhibit a 5 nt spacer, implying that ESR1 may be capable of binding to sites that were not previously detected. The symmetry between the half sites exhibited by the bipartite iPWMs suggests that dimeric ESR1 may bind a narrow range of sequences with similar half site affinities.
- The palindromic predominant sequence of the AP2 family is 5΄-GCCN_3_GGC-3΄, and other binding sequences confirmed in an *in vitro* binding-site selection assay include 5΄-GCCN_4_GGC-3΄ and 5΄-GCCN_3/4_GGG-3΄. Another binding site 5΄-CCCCAGGC-3΄ was also found in the SV40 enhancer (23). The spacer lengths in the bipartite iPWMs for AP2A and AP2C range from 2 to 4 nt, which is representative of the genome-wide pool of true binding sites (Figure 2B). We also noted that the two outermost positions are the most variable, and that adenine (instead of the consensus guanine) can also appear at the first position of the right half site. These bipartite iPWMs exhibit similar conservation levels across all the individual positions, suggesting that these binding sites of the two AP2 members may exhibit similar degrees of binding affinity, though iPWMs can recognize different sequences.
- The predominant spacer length separating half sites recognized by STAT1 is 3 nt; however, previous reports describe sites with a 2 nt gap, but not those separated by 4 nt (24). However, the STAT1 bipartite iPWM is based on 1709 binding sites (∼18%) with a 4 nt spacer, with most half sites separated by 2 or 3 nt (Figure 2B). The left- and rightmost nucleotides are nearly invariant, whereas the inner 2 nt contacts in each half site are variable.
- NFE2 and BACH1 heterodimerize with the MAF family (MAFF, MAFG and MAFK), and recognize two types of bipartite palindromic motifs, defined by the predominant binding sites TGCTGA(C)TCAGCA and TGCTGA(CG)TCAGCA (25). The previously reported binding motifs (3) are contiguous, and do not account for the dimeric interaction that gives rise to this bipartite binding pattern. The bipartite iPWMs indicate that the inner six positions surrounding the dominant 1 nt spacer exhibit higher information contents than the outer six positions (Figure 2B).

#### Comparing iPWMs for the same TF in distinct cell lines

**Figure 3.**
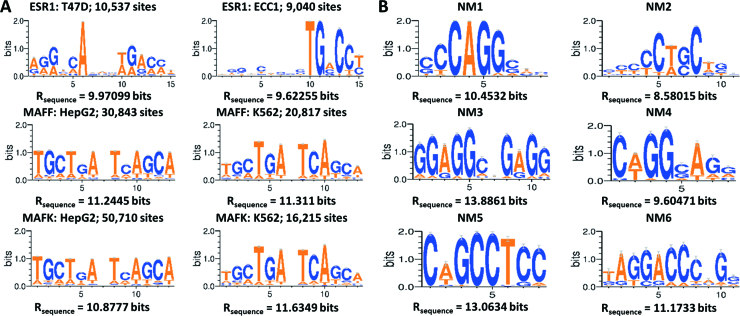
Comparison between iPWMs from different cell lines and novel motifs. (**A**) Each row includes sequence logos of two iPWMs of the same TF from two different cell lines. The bipartite iPWMs for MAFF and MAFK used the search pattern 6<1,2>6. (**B**) The high-confidence novel motifs (‘NM1’ – ‘NM6’). The logos of the NM1, NM2 and NM3 motifs come from the datasets of BAF155, NANOG and ESRRA, respectively.

Cell-type-specific differences between iPWMs of the same TF were evident for certain contiguous and bipartite motifs. For instance, among the three contiguous iPWMs of ESR1 derived from the ECC1 steroid-responsive endometrial cell line, conservation levels in the respective half sites are asymmetric, whereas the average information of these half sites are much more symmetric in iPWMs derived from T47D, a breast tumor cell line (Figure 3A). For the TFs MAFF and MAFK, the discrepancy between the bipartite iPWMs from K562 and HepG2 cells is evident: the outer six positions show a greater degree of conservation than the internal six positions in HepG2, but in K562 the opposite trend is illustrated (Figure 3A). The MAFK iPWM derived from ChIP-seq data of IMR90 cells resembles the HepG2 iPWMs, whereas the iPWMs from HeLa-S3 and H1-hESC datasets resemble the K562 iPWMs. The compositions of binding sites (i.e. different target genes for the same TF in different tissues) account for these differences because TFs can display distinct cell-type-specific DNA sequence preferences (26). Consistent iPWMs between replicate datasets makes it unlikely that the skewed base conservation between ChIP-seq datasets for the same TF in different cell lines arises from sampling differences; however, this possibility cannot be excluded.

### Cofactor binding motifs

**Figure 4.**
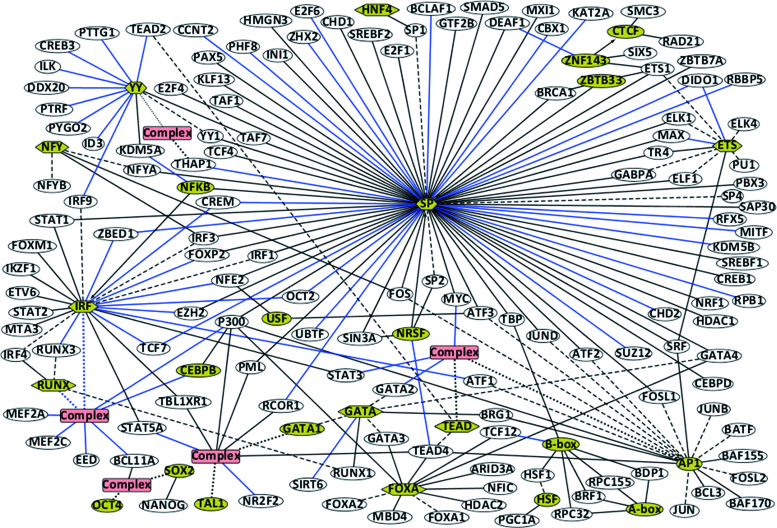
Network graph of TF-cofactor interactions revealed by the Maskminent pipeline. A yellow ellipse denotes a cofactor and a white ellipse denotes a primary TF. A hexagon denotes a TF family with dash lines connecting its members. For a TF family only members for which ENCODE provides peak datasets are shown. A red rectangle denotes a known or predicted TF complex with black or blue dotted lines indicating its components, respectively. An undirected line denotes the interaction between a primary TF and a cofactor which may be a complex or a TF family. A directed line links two cofactors, denoting that in a dataset of the starting TF, the ending TF was discovered as a cofactor. Black lines denote known interactions and blue lines denote the newly discovered interactions.

Discovery of the binding motif of a cofactor in the same ChIP-seq dataset for a primary TF implies that the two TFs transcriptionally co-regulate this set of common target genes. This could be accomplished either by formation of a physical complex on the promoter, or by synergistic or antagonistic *cis*-regulatory effects. *De novo* motif discovery from ChIP-seq datasets provides an effective approach for confirming or predicting statistically significant TF interactions on a genome-wide scale; by contrast, the abundant, existing literature overwhelmingly documents gene-by-gene evidence about such interactions which constrains arguments supporting their generalizability. Figure 4 illustrates TF-cofactor interactions revealed by the Maskminent pipeline.

#### Confirmation of known cofactors

The derived iPWMs confirmed genome-wide interactions between 22 cofactors and 102 primary TFs (Table 1), which were supported by the previous studies (3,5,6,15,27–93). For example, the interaction between SP1 and multiple members of the ETS and AP1 families has been well characterized (94–99). ELK1 and SRF can recruit each other to form a ternary complex on CArG-ETS elements (100). TEAD-AP1 cooperation with steroid receptor coactivators (SRC) drives downstream gene transcription to regulate cancer cell migration and invasion (101), and STAT1, STAT2 and IRF9 form a heterotrimer that regulates transcription of genes containing IFN-stimulated response elements (102). Consistent with previous reports (15), the existence of a YY1–THAP1 complex is predicted from co-segregation of their binding motifs in the K562 dataset of THAP1. Similarly, we predict that the SOX2–OCT4 complex colocalizes with BCL11A, similar to Wang *et al.* (3). A DNA-binding complex consisting of GATA1, TAL1, E2A, LMO2 and LDB1 is present in the erythroid cell lineage (103). Based on the proximity and coprecipitation of these binding sequences, we and others (3,104) find that this complex, in which GATA1 and TAL1 contact DNA, coordinately binds with TEAD4 and other non-DNA binding proteins (P300, PML, RCOR1 and TBL1XR1). The GATA1–TAL1 and SOX2–OCT4 complexes emerged from the datasets of TAL1 and OCT4 as primary motifs, respectively, which implies the formation of the two complexes may be necessary for binding of TAL1 and OCT4.

**Table 1.** Cofactors revealed by iPWMs and their corresponding primary TFs

Cofactors	Primary TFs*	
	Sequence-specific	Non-sequence-specific
AP1	GATA2, MYC, SRF, STAT3, TEAD4	BAF155, BAF170, BCL3, BRG1, P300
CEBPB		P300
CTCF	ZNF143	RAD21, SMC3
ETS family	MAX, SRF^1^, TR4	DIDO1^2^
GATA family	RUNX1 ^2^	BRG1 ^2^, SIRT6^2^
GATA1–TAL1	NR2F2^2^, STAT5A^2^, TAL1^2^, TEAD4^2^	P300 ^2^, PML^2^, RCOR1^2^, TBL1XR1^2^
FOXA family	ARID3A ^3^, GATA3, GATA4^3^, NFIC^3^, TCF12^3^, TEAD4^3^	HDAC2 ^3^, MBD4^3^, P300
HNF4 family	SP1 ^3^	
HSF family		PGC1A ^3^
IRF family	ATF1^2^, BCL11A^1^, CEBPB^1^, CREM^1^, ETV6^1^, FOXM1^1^, FOXP2, IKZF1^1^, MEF2A^1^, MEF2C^1^, NFE2^1^, NFKB^1^, OCT2^1^, RUNX3^1^, STAT1^2^, STAT2^2^, STAT3^1^, STAT5A^1^, TCF7^1^, ZBED1^1^	EED^1^, EZH2^1^, MTA3^1^, P300^1^, TBL1XR1^1^
NFKB		KDM5A^4^
NFY	FOS, IRF3	
NRSF	SP2 ^3^, TEAD4	SIN3A ^4^
RUNX family	BCL11A ^1^, CEBPB^1^, IRF4^1^, MEF2A^1^, MEF2C^1^	EED ^1^, P300^1^
SP family	ATF2 ^4^, ATF3, CEBPD^3^, CREB1, CREM^1^, DEAF1^2^, E2F1, E2F4, E2F6, ELF1, ELK1, ETS1, FOS, FOSL1^4^, FOXP2, GABPA, GATA4^3^, IRF1^2^, IRF3, JUND, KLF13^2^, MAX, MITF^2^, MXI1, MYC, NFE2^1^, NFKB^1^, NFYA, NRF1, NRSF^3^, OCT2^1^, PAX5^1^, PBX3, RFX5, SMAD5, SREBF1^3^, SREBF2^3^, SRF, STAT1^1^, SUZ12, TBP, TCF4, TCF7^2^, THAP1^2^, TR4, UBTF^2^, YY1, ZBED1^2^, ZBTB33, ZBTB7A^2^, ZHX2^3^	BCLAF1, BRCA1, CBX1^3^, CCNT2^2^, CHD1, CHD2, DIDO1^2^, EZH2, GTF2B^2^, HDAC1^2^, HMGN3^2^, INI1, KAT2A, KDM5B^2^, P300^4^, PHF8^2^, PML, RBBP5, RCOR1^3^, RPB1, SAP30^2^, SIN3A, TAF1, TAF7
SOX2	NANOG ^4^	
SOX2-OCT4	BCL11A ^4^, OCT4^4^	
TEAD family	GATA2, MYC, STAT3	
TFIIIC	HSF1 ^3^, TBP, TCF12	BDP1, BRF1, RPC155, RPC32
YY family	CREB3^2^, IRF9^2^, PTTG1^2^, TEAD2^2^, THAP1^2^	DDX20^2^, ID3^2^, ILK^2^, KDM5A^4^, PTRF^2^, PYGO2^2^, TAF7^2^
USF	ATF3, NFE2^1^	
ZBTB33	ETS1 ^1^	BRCA1
ZNF143	ETS1, DEAF1^2^	SIX5

*The underlined or normal font denotes known or newly discovered interactions between cofactors and primary TFs, respectively.

^1,2,3,4^The cofactor was revealed in the GM12878-related, K562, HepG2 or H1-HESC cell lines, respectively. Otherwise the cofactor appeared in other or multiple cell lines.

#### Discovery of novel cofactors

Maskminent revealed a number of previously unrecognized cofactor motifs (*n* = 10) for 46 primary TFs (Table 1), which supports novel TF cobinding and interactions. This includes possible associations between the IRF and RUNX families, and their further cooperation with BCL11A, MEF2A, MEF2C, CEBPB, EED and P300 in GM12878 cells (Table 1 and Figure 4). Similarly, the TEAD–AP1 complex is predicted to recruit MYC, STAT3 and GATA2 in multiple cell lines. The finding that NR2F2 and STAT5A motifs are in close proximity to sequences recognized by the GATA1–TAL1 complex suggests these factors may coordinately regulate target genes. Many cofactors were also discovered among datasets of non-sequence-specific primary TFs, which is consistent with the possibility that these primary TFs are recruited to gene promoters through their association with DNA-binding cofactors (Table 1).

#### Cofactor binding sites

**Figure 5.**
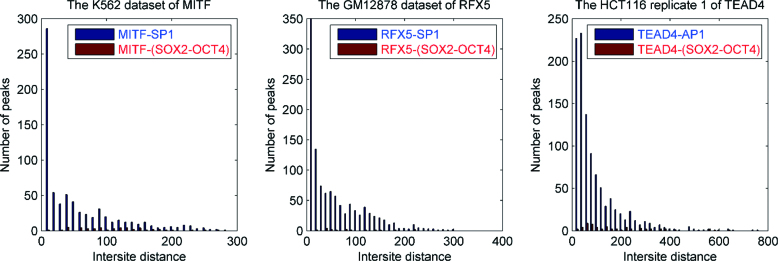
Distributions of intersite distances between primary TFs and discovered cofactors versus negative controls. The minimum threshold on information contents of predicted binding sites is *R*_sequence_. Each graph illustrates a much higher frequency of short (<20 nt) intersite distances between primary TFs and cofactors (blue) compared to the negative control (SOX2-OCT4; red).

To validate the predicted cobinding between cofactors and primary TFs, we determined the intersite distance distributions by scanning the individual ChIP-seq intervals with the derived iPWMs for each (Figure 5 and Supplementary Data). A minimum information threshold was applied to the *R_i_* values of predicted binding sites in order to remove the relatively large number of weak binding sites that are likely to be low-complexity sequences (e.g. *R*_sequence_ [or 0.5 * *R*_sequence_, if too many cofactor binding sites were eliminated at the higher threshold]). The SOX2–OCT4 complex was used as a primary negative control, as it is primarily expressed in the H1-hESC cell line and is unlikely to be a cofactor for primary TFs in other cell lines. A large percentage of peaks have short intersite distances between the primary TF and the corresponding cofactor binding sites (e.g. <20 nt), whereas there is no such a trend for the negative control sequences and the primary TF. The same difference is observed between the distribution for the documented TEAD4–AP1 binding site pair and for the negative control set. Consistent with previous reports (4), the binding sites of cofactors and primary TFs in peak datasets were physically overlapped between the IRF and RUNX motifs, between the TEAD4 and AP1 motifs, and between USF and ATF3 (AP1) recognition motifs.

#### Tissue-specific preferences of predicted cofactors relative to primary TFs

Several cofactors were recurrently associated with different primary TF partners, notably in specific cell lines. One possible explanation is that these cofactors are coordinately regulated with different primary TFs preferentially in specific cell types. For example, the datasets of 25 primary TFs in which the IRF family was discovered as a cofactor were all derived from lymphoblastoid (e.g. GM12878) cell lines, with four exceptions (Table 1). Regulation by the IRF family is central to B-lymphocyte expression programs (105). All the datasets of 11 primary TFs from which the GATA and GATA1–TAL1 motifs emerged as cofactors were derived from K562 erythrocytic leukemia cells (Table 1), which is consistent with the activation role that the GATA family exhibits in hematopoietic lineage gene expression (106,107). Similarly, FOXA family members bind to the same sequences as seven primary TFs in the HepG2 cell line derived from hepatocellular carcinoma cells (Table 1), which is consistent with the fact that FOXA proteins regulate the initiation of liver development (108). Datasets of GATA3 and P300 from the T47D breast cancer cell line are also linked to FOXA. Another TF family known to be a key factor regulating hepatocyte differentiation and liver-specific functions is HNF4 (109), which was discovered as a cofactor of SP1 in a HepG2 dataset. SOX2 and the SOX2–OCT4 complex were unveiled as cofactors only in datasets of three primary TFs from the H1-hESC cell line representing embryonic stem cells (Table 1), which is supported by the requirement for SOX2, OCT4 and NANOG to maintain pluripotency (110). Interestingly, all the datasets (*n* = 12) in which YY was revealed as a cofactor were from K562 cells, with one exception (Table 1). Unlike the GATA TFs, the YY family is ubiquitously distributed and not known to play an especially central role in erythroid lineage development, although YY1 is known to act as a developmental repressor of the ε-globin gene along with GATA1 (111).

Not surprisingly, the SP family was found to be capable of interacting with the maximum number of TFs, which is consonant with its role in constitutive transcriptional activation. Similarly, the ubiquitously expressed AP1 interacts with 10 TFs in multiple cell lines, and these interactions do not show any preference in cell type.

A number of primary TFs exhibit an extensive capability of interacting with multiple cofactors in different tissues. The unique distribution of these cofactors across multiple cell lines suggests the tissue-specific functions of the primary TFs. For instance, TEAD4 was found to coimmunoprecipitate with GATA1–TAL1 in K562 cells, NRSF in A549 cells, FOXA in HepG2 cells, and AP1 in multiple cell types. Cofactors of P300 include IRF–RUNX in GM12878 cells, SP in H1-hESC cells, AP1 and CEBPB in HeLa-S3 cells, FOXA in HepG2 and T47D cells and GATA1–TAL1 in K562 cells. Cosegregation analysis revealed interactions between BCL11A and IRF–RUNX in GM12878 cells, and SOX2-OCT4 in H1-hESC cells. STAT5A and TBL1XR1 cosegregated with members of the IRF family in GM12878 cells and with GATA1–TAL1 in K562 cells.

#### Discordance between iPWMs derived from the same ChIP-seq assay

We noticed some discrepancies between IDR-thresholded datasets and SPP datasets from the same ChIP-seq assay. For example, for the primary TF BRG1, iPWMs exclusively from SPP-derived datasets exhibit motifs of GATA1 and AP1; IDR-thresholded BRG1 data produced only noisy low information content motifs. We also noticed that the motifs derived from different biological replicates of the same ChIP-seq assay were sometimes inconsistent. One replicate of the TEAD4 ChIP-seq assay from the A549 cell line revealed only the NRSF binding motif, whereas both the cofactor AP1 and the primary motif were derived from the other replicate.

### Novel binding motifs

We uncovered six high-confidence novel motifs that have not been previously annotated (Figure 3B). The ‘NM1’ motif was considerably enriched in the datasets of BAF155 and BRG1 (which do not bind DNA directly) from HeLa-S3 cells and the ‘NM2’ motif was highly conserved in the datasets of BCL11A and NANOG from H1-hESC cells. The ‘NM3’ motif was revealed in the ESRRA and SREBF2 datasets from GM12878 cells, in the MAX dataset from HCT116, in the CREB1 and GTF3C2 datasets from K562, and in the non-DNA-binding RCOR1 dataset from IMR90 cells. The Euclidean distances between these novel motifs and primary motifs are dissimilar, ranging from 3.1 to 3.4 bits/nt. The ‘NM4’, ‘NM5’ and ‘NM6’ motifs were discovered in the datasets of GATA3, MXI1 and FOSL1 from MCF-7, SK-N-SH and H1-hESC cells, respectively, with distances ranging from 2.9 to 3.4 bits/nt.

We investigated whether these novel motifs were enriched in hallmarks of open chromatin, based on the co-occurrence with DNase I hypersensitive sites and near H3K4me and H3K27ac histone modifications (112). After scanning the complete genome with these iPWMs, the proportions of sites detected within these corresponding ENCODE chromatin tracks were determined for the respective cell lines (Table 2). These proportions (5–35%) are consistent with previously reports of binding sites for other TFs (113). The frequencies of sites detected with the NM2 and NM6 motifs within the H3K4me1 and H3K27ac peaks are significantly higher than those found after intersection of each NM binding site with the H3K4me2 and H3K4me3 tracks, respectively. The co-occurence of NM2 and NM6 with the H3K4me1 and H3K27ac epigenetic marks supports the assignment of these motifs as components of transcriptional enhancer elements, because these histone modifications are present in nucleosomes flanking enhancer elements (114). Additionally, the co-occurence of these two motifs within DNase I hypersensitive intervals exhibit the highest among all the six motifs. The remaining motifs could represent binding motifs of currently unknown TFs or other non-annotated functional elements.

**Table 2.** Percentages of binding sites from novel motifs (NM) that overlap DNase I hypersensitive intervals and/or regions of specific histone modifications

	ENCODE Genome Browser Track				
Novel motif	DNase I HS	H3K4me1	H3K4me2	H3K4me3	H3K27ac
NM1^†^	4.50%	17.63%	15.52%	16.23%	11.44%
NM2^†^	7.06%	33.63%	14.39%	9.61%	34.05%
NM3^†^	4.21%	21.19%	16.89%	13.75%	12.25%
NM4	3.18%	N/A*	N/A*	1.04%	2.22%
NM5	2.31%	N/A*	N/A*	1.21%	N/A*
NM6	6.16%	32.37%	13.58%	9.36%	34.10%

*The histone modification data for the specific cell line used to derive the iPWM is unavailable.

^†^The iPWMs of the NM1, NM2 and NM3 motifs used to scan the hg19 genome assembly come from the datasets of BAF155, NANOG and ESRRA, respectively.

### Binding site motif validation

#### Detection of true binding sites with iPWMs

A total of 803 experimentally-confirmed, previously published binding sites were verified for the 93 TFs whose primary binding motifs had been identified (Supplementary Data). We detected these sites with the derived iPWMs by scanning promoters of known TF target genes for binding elements with positive *R_i_* values. There was complete concordance between these true binding sites and those detected with the iPWMs, both in terms of their locations and relative strengths. For example, an electrophoretic mobility shift assay analysis of the SERPINA3 promoter proved that the nucleotide sequence starting at GRCh38 (chr14:94612260) contains a stronger binding site of STAT1 than the one starting at GRCh38 (chr14:94612291) (Supplementary Data) (115); the binding site (5΄-TTCTGGTAA-3΄ with *R_i_* = 9.02 bits; Row 781) detected by the bipartite iPWM is indeed 2^2.13^ (or 4.38)-fold stronger than the other site (5΄-TTCTCGGA-3΄ with *R_i_* = 6.89 bits; Row 782) detected in this promoter.

#### Correspondence between functionally characterized SNPs and changes in information content

Based on the change in the *R_i_* value of a binding site, the effect of a SNP on the binding site strength can be predicted with iPWMs (10,12). For 153 SNPs within the binding sites of 29 TFs, we determined *R_i_* values of the variant sequence for the corresponding iPWM and compared the predicted consequence to observed TF binding, and if available, published changes in expression (Supplementary Data). For 130 SNPs (∼85.0%) affecting binding sites of 27 TFs, the predictions of the iPWMs and the experimental observations are completely concordant. For 16 SNPs (∼10.5%) affecting binding sites of 10 TFs, the predicted and observed experimental findings are concordant, but the extents of these changes differ (e.g. TF binding is predicted to only be weakened, but binding or expression was completely abolished). For 7 SNPs (∼4.6%) altering binding sites of three TFs, the predicted and observed experimental changes were discordant. iPWMs for two (CEBPB and SP1) of these three TFs were validated for other SNPs.

#### Comparison between iPWMs and other binding motifs

Binding motifs of eukaryotic TFs in the CIS-BP database were previously reconstructed from oligonucleotide binding selection assays (13); these motifs represent another type of ground truth reflecting the genuine sequence preferences of these TFs. For 133 TFs, we quantitatively compared the iPWMs with these motifs by determining the normalized Euclidean distances between them, and classified the distances into three categories. We observed that the iPWMs derived in this study and the reconstructed motifs are nearly identical (<1 bit/nt) for 75 TFs, or only differ at 1 or 2 positions (1-2 bits/nt) for 18 TFs. The discovery of cofactors was the predominant explanation for large distances (>2 bits/nt) for 39 of these TFs.

#### Statistical analyses on iPWMs

**Figure 6.**
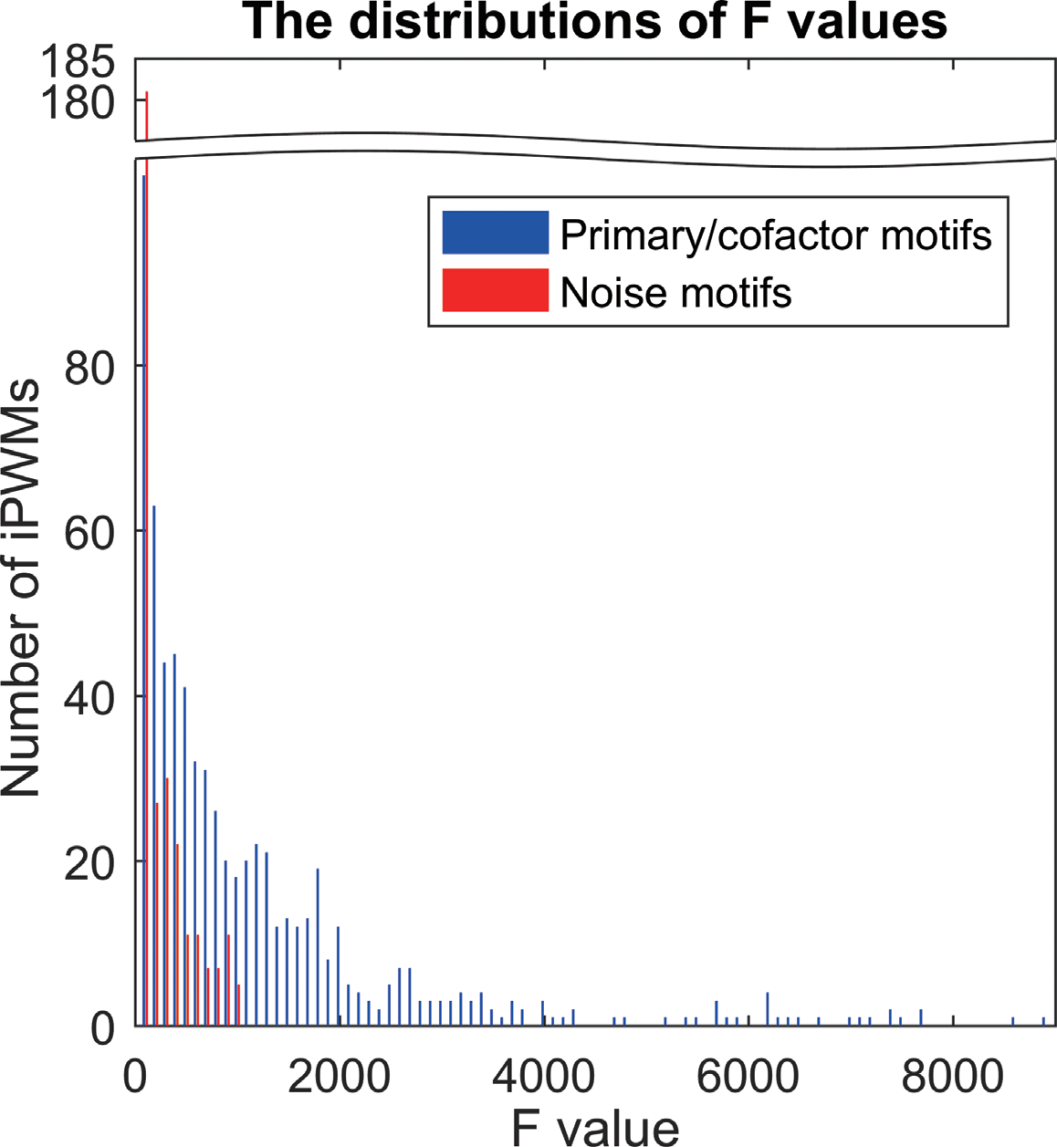
F-test results evaluating the relationship between *R_i_* values and binding energy. The proportion of *F*-values within the first bin for primary/cofactor motifs is much higher than that for noise motifs. A minimum threshold of 1000 correctly classifies all the noise motifs and 37.2% (251/674) of primary/cofactor motifs.

To distinguish true binding motifs from noise motifs, the relationship between *R_i_* values and binding energy was evaluated by performing F tests on all binding sites in all of the contiguous iPWMs that we derived (674 primary/cofactor, 312 noise). The *F*-values are plotted as a histogram to illustrate probability density distributions (Figure 6; data available in Supplementary Data). The histogram shows that most *F*-values between 0 and 100 were significantly enriched for noise motifs. In general, the *F*-values of primary/cofactor motifs significantly exceed those derived from noise. The primary/cofactor motif and noise motif distributions are different (Mann–Whitney U test; *P* = 3.1E-57 at 1% significance level). We note that only primary and cofactor motifs exhibit *F*-values > 1000, which comprise 37.2% (251 of 674) of all iPWMs. The iPWMs with *F*-values < 1000 remain valid based on the other criteria described above.

## DISCUSSION

In this study, we derived and validated TF binding motifs from ChIP-seq datasets using an information theory-based approach, also revealing TF cofactor binding sites and other novel motifs. The primary TF motifs were validated by comparison with motifs derived independently from binding studies, by analysis of gene variants known to alter TF binding affinities, and by comparing the locations of binding sites predicted by iPWMs with those of true sites previously determined in published binding and expression studies. In addition to contiguous iPWMs, bipartite iPWMs with variable-length spacers were also derived. These iPWMs more precisely reflect the binding behavior of dimeric TFs, as they incorporate intermediate and often weak binding sites that are often excluded from consensus sequence-based (strong) binding site sets (3). This enables these iPWMs to accurately quantify binding site strengths across a broad range of affinities (Supplementary Data). To test this, the iPWMs were applied to mutation analyses of regulatory SNPs (Supplementary Data). We have recently used this approach to identify and prioritize variants affecting TF binding in 20 risk genes of 287 hereditary breast and ovarian cancer patients (116) and 7 genes from 102 such patients (117). In present study, the iPWMs were also used to delineate known and novel TF-cofactor interactions.

TF binding sites across the genome have been predicted from promoter accessibility analyses with high-throughput DNase-seq assays. For each of 20 TFs, Yardımcı *et al.* (118) obtained a set of true binding sites by intersecting ChIP-seq peaks with the 50 000 strongest binding sites predicted by JASPAR and TRANSFAC PWMs in the genome. The FLR (Footprint Log-likelihood Ratio), which is defined as the logarithm of the ratio between probabilities that a DNase I footprint is produced by either a true binding site or a background sequence, was determined at these sites. We attempted to detect these true sites using the derived iPWMs. For these 20 TFs, all of these sites (ranging from *n* = 31 to 21 550, depending on the TF) were successfully detected by the iPWMs (*R_i_* > 0). By contrast, the FLR identified 35–85% of the verified binding sites (Supplementary Data). As weak binding sites tend not to generate footprints and thus not to be discovered by DNase-seq, the expectation is that the sites detected by DNase-seq would be stronger than those that evade detection. In fact, this trend was observed for only 10 TFs and the average strengths of these classes of these binding sites were not significantly different.

In the Maskminent pipeline, the weak peaks below the threshold signal intensity do not necessarily contain weak or are missing binding sites; in fact, the distribution of *R_i_* values of binding sites in these bottom peaks is similar to that in the top peaks used to derive the iPWM (Supplementary Data). Thresholding the dataset is required in order to ensure that the iPWM for the primary motif consists of binding sites from as many peaks as possible, while preventing alternative motifs from dominating the objective function used in Maskminent.

We also compared results produced by the Maskminent pipeline with other motif discovery tools from two perspectives of revealing primary and cofactor binding motifs (Supplementary Data). MEME-ChIP was previously used to derive motifs for 457 ChIP-seq datasets (119) and SeqGL (120) was used to analyze 105 datasets. Among the sequence-specific TFs (*n* = 98) investigated by both tools, Maskminent and MEME-ChIP discovered primary motifs for 80 (∼81.6%) and 92 (∼93.9%) TFs, respectively. Among the 59 TF datasets analyzed by Maskminent, MEME-ChIP, SeqGL and HOMER (121), primary motifs were revealed for 45 (∼76.3%), 51 (∼86.4%), 49 (∼83.1%) and 47 (∼79.7%) datasets, respectively. The cofactor motifs that Maskminent found (which MEME-ChIP and SeqGL failed to detect) primarily comprise the SP family. Since MEME and SeqGL discriminate binding sites from background sequences using nucleotide frequencies computed from all input sequences, binding motifs with compositions similar to the background may fail to be discovered, such as the SP motif; in contrast, Maskminent does not rely on background compositions and will always return the lowest entropy motif. While MEME-ChIP and SeqGL revealed a greater number of cofactor motifs, selecting only the top 500 or 2000 peaks increases the likelihood that those cofactors appeared by chance. This is because MEME-ChIP and SeqGL were configured to report multiple motifs, whereas the main objective of Maskminent was to discover primary motifs (i.e. if the initial iPWM derived from a dataset exhibits the primary motif, the masking and thresholding techniques will no longer be used, unless it is explicitly masked). Finally, the ability of Maskminent, MEME-ChIP, SeqGL to reveal binding motifs was compared on the 105 datasets (120). Each tool discovers cofactor motifs that others do not recognize.

Arvey *et al.* (26) trained support vector machines (SVMs) that use flexible *k*-mer patterns to capture DNA sequence signals more accurately from 286 ChIP-seq experiments than traditional motif approaches, and these SVMs can also integrate histone modifications and DNase accessibility to significantly more accurately predict TF occupancy than simpler approaches. However, the SVM approach does not provide any insight into binding strength. Even though accessibility constrains the number of binding sites and increases the accuracy of binding site detection, it is not possible to compare binding site strengths once the designated sites are combined with DNase I hypersensitivity profiles and other chromatin accessibility marks.

In fact, the number of TFs for which cofactor motifs were revealed exceeds the number of TFs whose primary binding motifs were discovered, partially because only cofactor motifs can be found in the datasets of TFs which exhibit little or no sequence specificity (e.g. CCNT2, INI1 and P300). For 11 primary TFs, the binding site sequences were extremely variable; that is, the overall conservation levels of their binding motifs contain less information than noisy, low complexity sequences or cofactor motifs. For 18 primary TFs associated with cofactors, which themselves physically contact DNA, the primary TF motif was not enriched. The inability of the software to discover such primary motifs is a limitation of this approach. Interactions between the primary TFs and a subset of the cofactors which are known to cooperate with them were detected, since the association has to occur with a prevalence sufficient to produce a recognizable motif (usually >0.5 bit/nt over the entire site). Nevertheless, the algorithm may not find cofactors with weakly conserved motifs or those that overlap with other conserved motifs.

While unable to discover cofactors nor identify bipartite motifs of variable spacing, the oligonucleotide microarray technique adopted by Weirauch *et al.* (13) and Jolma *et al.* (14) theoretically is able to determine binding specificities for all the sequence-specific TFs, because contiguous binding sites of TFs are reconstructed from overlapping oligonucleotide sequences by directly detecting complexes with the TF. This eliminates interference of noisy sequences or cofactors which may emerge as false minimum entropies using our method.

The Maskminent pipeline can be applied to other ChIP-seq data not included in ENCODE. The quality control criteria we described are capable of ensuring that the user-built iPWMs are accurate and can be used for binding site detection. The first and second criteria are particularly important, because they provide a straightforward assessment of iPWM performance. The recursively thresholded feature is crucial for guaranteeing that the discovered cofactors do not appear by chance, because the greater the number of peaks from which a cofactor is derived, the higher the confidence that the cofactor indeed interacts with the primary factor.

In summary, we comprehensively investigated and implemented a new approach to define TF binding specificities based on the ChIP-seq TF data that ENCODE has released. This allowed us to mine and quantify both known and previously unrecognized TF binding motifs and cofactor interactions on a genome scale. This information expands the granularity of the current knowledge on TF interaction with DNA and points out potential directions for future experimental study on interaction between TFs.

## SOFTWARE AVAILABILITY

http://dx.doi.org/10.5281/zenodo.49234 and https://www.mutationforecaster.com.

## Supplementary Material

Supplementary Data

## References

[1] Leung K.K., Ng L.J., Ho K.K., Tam P.P., Cheah K.S. Different cis-regulatory DNA elements mediate developmental stage- and tissue-specific expression of the human COL2A1 gene in transgenic mice. J. Cell Biol. 1998; 141:1291–1300. 9628886 10.1083/jcb.141.6.1291 PMC2132792

[2] Levine M., Tjian R. Transcription regulation and animal diversity. Nature. 2003; 424:147–151. 12853946 10.1038/nature01763

[3] Wang J., Zhuang J., Iyer S., Lin X., Whitfield T.W., Greven M.C., Pierce B.G., Dong X., Kundaje A., Cheng Y. Sequence features and chromatin structure around the genomic regions bound by 119 human transcription factors. Genome Res. 2012; 22:1798–1812. 22955990 10.1101/gr.139105.112 PMC3431495

[4] Jolma A., Yin Y., Nitta K.R., Dave K., Popov A., Taipale M., Enge M., Kivioja T., Morgunova E., Taipale J. DNA-dependent formation of transcription factor pairs alters their binding specificity. Nature. 2015; 527:384–388. 26550823 10.1038/nature15518

[5] Parelho V., Hadjur S., Spivakov M., Leleu M., Sauer S., Gregson H.C., Jarmuz A., Canzonetta C., Webster Z., Nesterova T. Cohesins functionally associate with CTCF on mammalian chromosome arms. Cell. 2008; 132:422–433. 18237772 10.1016/j.cell.2008.01.011

[6] Fleming J.D., Pavesi G., Benatti P., Imbriano C., Mantovani R., Struhl K. NF-Y coassociates with FOS at promoters, enhancers, repetitive elements, and inactive chromatin regions, and is stereo-positioned with growth-controlling transcription factors. Genome Res. 2013; 23:1195–1209. 23595228 10.1101/gr.148080.112 PMC3730095

[7] ENCODE Project Consortium. An integrated encyclopedia of DNA elements in the human genome. Nature. 2012; 489:57–74. 22955616 10.1038/nature11247 PMC3439153

[8] Bi C., Rogan P.K. Bipartite pattern discovery by entropy minimization-based multiple local alignment. Nucleic Acids Res. 2004; 32:4979–4991. 15388800 10.1093/nar/gkh825 PMC521645

[9] Shannon C.E. A mathematical theory of communication. Bell Syst. Technol. J. 1948; 27:379–423.

[10] Schneider T.D. Information content of individual genetic sequences. J. Theor. Biol. 1997; 189:427–441. 9446751 10.1006/jtbi.1997.0540

[11] Schneider T.D. Measuring molecular information. J. Theor. Biol. 1999; 201:87–92. 10534438 10.1006/jtbi.1999.1012

[12] Rogan P.K., Faux B.M., Schneider T.D. Information analysis of human splice site mutations. Hum. Mutat. 1998; 12:153–171. 9711873 10.1002/(SICI)1098-1004(1998)12:3<153::AID-HUMU3>3.0.CO;2-I

[13] Weirauch M.T., Yang A., Albu M., Cote A.G., Montenegro-Montero A., Drewe P., Najafabadi H.S., Lambert S.A., Mann I., Cook K. Determination and inference of eukaryotic transcription factor sequence specificity. Cell. 2014; 158:1431–1443. 25215497 10.1016/j.cell.2014.08.009 PMC4163041

[14] Jolma A., Yan J., Whitington T., Toivonen J., Nitta K.R., Rastas P., Morgunova E., Enge M., Taipale M., Wei G. DNA-binding specificities of human transcription factors. Cell. 2013; 152:327–339. 23332764 10.1016/j.cell.2012.12.009

[15] Kheradpour P., Kellis M. Systematic discovery and characterization of regulatory motifs in ENCODE TF binding experiments. Nucleic Acids Res. 2014; 42:2976–2987. 24335146 10.1093/nar/gkt1249 PMC3950668

[16] Schneider T.D. Consensus sequence Zen. Appl. Bioinformatics. 2002; 1:111–119. 15130839 PMC1852464

[17] Landt S.G., Marinov G.K., Kundaje A., Kheradpour P., Pauli F., Batzoglou S., Bernstein B.E., Bickel P., Brown J.B., Cayting P. ChIP-seq guidelines and practices of the ENCODE and modENCODE consortia. Genome Res. 2012; 22:1813–1831. 22955991 10.1101/gr.136184.111 PMC3431496

[18] Wang J., Zhuang J., Iyer S., Lin X.-Y., Greven M.C., Kim B.-H., Moore J., Pierce B.G., Dong X., Virgil D. Factorbook.org: a Wiki-based database for transcription factor-binding data generated by the ENCODE consortium. Nucleic Acids Res. 2013; 41:D171–D176. 23203885 10.1093/nar/gks1221 PMC3531197

[19] Kharchenko P.V., Tolstorukov M.Y., Park P.J. Design and analysis of ChIP-seq experiments for DNA-binding proteins. Nat. Biotechnol. 2008; 26:1351–1359. 19029915 10.1038/nbt.1508 PMC2597701

[20] Bailey T.L., Boden M., Buske F.A., Frith M., Grant C.E., Clementi L., Ren J., Li W.W., Noble W.S. MEME SUITE: tools for motif discovery and searching. Nucleic Acids Res. 2009; 37:W202–W208. 19458158 10.1093/nar/gkp335 PMC2703892

[21] Tanay A. Extensive low-affinity transcriptional interactions in the yeast genome. Genome Res. 2006; 16:962–972. 16809671 10.1101/gr.5113606 PMC1524868

[22] El Marzouk S., Gahattamaneni R., Joshi S.R., Scovell W.M. The plasticity of estrogen receptor-DNA complexes: binding affinity and specificity of estrogen receptors to estrogen response element half-sites separated by variant spacers. J. Steroid Biochem. Mol. Biol. 2008; 110:186–195. 18479910 10.1016/j.jsbmb.2008.03.034

[23] Eckert D., Buhl S., Weber S., Jager R., Schorle H. The AP-2 family of transcription factors. Genome Biol. 2005; 6:246. 16420676 10.1186/gb-2005-6-13-246 PMC1414101

[24] Ehret G.B., Reichenbach P., Schindler U., Horvath C.M., Fritz S., Nabholz M., Bucher P. DNA binding specificity of different STAT proteins. Comparison of in vitro specificity with natural target sites. J. Biol. Chem. 2001; 276:6675–6688. 11053426 10.1074/jbc.M001748200

[25] Kataoka K., Noda M., Nishizawa M. Maf nuclear oncoprotein recognizes sequences related to an AP-1 site and forms heterodimers with both Fos and Jun. Mol. Cell. Biol. 1994; 14:700–712. 8264639 10.1128/mcb.14.1.700-712.1994 PMC358419

[26] Arvey A., Agius P., Noble W.S., Leslie C. Sequence and chromatin determinants of cell-type-specific transcription factor binding. Genome Res. 2012; 22:1723–1734. 22955984 10.1101/gr.127712.111 PMC3431489

[27] Kawana M., Lee M.E., Quertermous E.E., Quertermous T. Cooperative interaction of GATA-2 and AP1 regulates transcription of the endothelin-1 gene. Mol. Cell. Biol. 1995; 15:4225–4231. 7623817 10.1128/mcb.15.8.4225 PMC230661

[28] Roca H., Pande M., Huo J.S., Hernandez J., Cavalcoli J.D., Pienta K.J., McEachin R.C. A bioinformatics approach reveals novel interactions of the OVOL transcription factors in the regulation of epithelial - mesenchymal cell reprogramming and cancer progression. BMC Syst. Biol. 2014; 8:29. 24612742 10.1186/1752-0509-8-29 PMC4008156

[29] Zhu C., Johansen F.E., Prywes R. Interaction of ATF6 and serum response factor. Mol. Cell. Biol. 1997; 17:4957–4966. 9271374 10.1128/mcb.17.9.4957 PMC232347

[30] Zhang X., Wrzeszczynska M.H., Horvath C.M., Darnell J.E. Interacting regions in Stat3 and c-Jun that participate in cooperative transcriptional activation. Mol. Cell. Biol. 1999; 19:7138–7146. 10490649 10.1128/mcb.19.10.7138 PMC84707

[31] Ito T., Yamauchi M., Nishina M., Yamamichi N., Mizutani T., Ui M., Murakami M., Iba H. Identification of SWI.SNF complex subunit BAF60a as a determinant of the transactivation potential of Fos/Jun dimers. J. Biol. Chem. 2001; 276:2852–2857. 11053448 10.1074/jbc.M009633200

[32] Na S.Y., Choi J.E., Kim H.J., Jhun B.H., Lee Y.C., Lee J.W. Bcl3, an IkappaB protein, stimulates activating protein-1 transactivation and cellular proliferation. J. Biol. Chem. 1999; 274:28491–28496. 10497212 10.1074/jbc.274.40.28491

[33] Henderson A., Holloway A., Reeves R., Tremethick D.J. Recruitment of SWI/SNF to the human immunodeficiency virus type 1 promoter. Mol. Cell. Biol. 2004; 24:389–397. 14673171 10.1128/MCB.24.1.389-397.2004 PMC303370

[34] Lee J.S., See R.H., Deng T., Shi Y. Adenovirus E1A downregulates cJun- and JunB-mediated transcription by targeting their coactivator p300. Mol. Cell. Biol. 1996; 16:4312–4326. 8754832 10.1128/mcb.16.8.4312 PMC231430

[35] Schwartz C., Beck K., Mink S., Schmolke M., Budde B., Wenning D., Klempnauer K.-H. Recruitment of p300 by C/EBPbeta triggers phosphorylation of p300 and modulates coactivator activity. EMBO J. 2003; 22:882–892. 12574124 10.1093/emboj/cdg076 PMC145436

[36] Bailey S.D., Zhang X., Desai K., Aid M., Corradin O., Cowper-Sal Lari R., Akhtar-Zaidi B., Scacheri P.C., Haibe-Kains B., Lupien M. ZNF143 provides sequence specificity to secure chromatin interactions at gene promoters. Nat. Commun. 2015; 2:6186. 25645053 10.1038/ncomms7186 PMC4431651

[37] O'Geen H., Lin Y.-H., Xu X., Echipare L., Komashko V.M., He D., Frietze S., Tanabe O., Shi L., Sartor M.A. Genome-wide binding of the orphan nuclear receptor TR4 suggests its general role in fundamental biological processes. BMC Genomics. 2010; 11:689. 21126370 10.1186/1471-2164-11-689 PMC3019231

[38] Elagib K.E., Racke F.K., Mogass M., Khetawat R., Delehanty L.L., Goldfarb A.N. RUNX1 and GATA-1 coexpression and cooperation in megakaryocytic differentiation. Blood. 2003; 101:4333–4341. 12576332 10.1182/blood-2002-09-2708

[39] Xu Z., Meng X., Cai Y., Koury M.J., Brandt S.J. Recruitment of the SWI/SNF protein Brg1 by a multiprotein complex effects transcriptional repression in murine erythroid progenitors. Biochem. J. 2006; 399:297–304. 16800816 10.1042/BJ20060873 PMC1609906

[40] Grau J., Grosse I., Posch S., Keilwagen J. Motif clustering with implications for transcription factor interactions. German Conf. Bioinformatics. 2015; doi:10.7287/peerj.preprints.1302v1.

[41] Albergaria A., Paredes J., Sousa B., Milanezi F., Carneiro V., Bastos J., Costa S., Vieira D., Lopes N., Lam E.W. Expression of FOXA1 and GATA-3 in breast cancer: the prognostic significance in hormone receptor-negative tumours. Breast Cancer Res. BCR. 2009; 11:R40. 19549328 10.1186/bcr2327 PMC2716509

[42] Cirillo L.A., Zaret K.S. An early developmental transcription factor complex that is more stable on nucleosome core particles than on free DNA. Mol. Cell. 1999; 4:961–969. 10635321 10.1016/s1097-2765(00)80225-7

[43] Grabowska M.M., Elliott A.D., DeGraff D.J., Anderson P.D., Anumanthan G., Yamashita H., Sun Q., Friedman D.B., Hachey D.L., Yu X. NFI transcription factors interact with FOXA1 to regulate prostate-specific gene expression. Mol. Endocrinol. Baltim. Md. 2014; 28:949–964. 10.1210/me.2013-1213 PMC4042066 24801505

[44] Kohler S., Cirillo L.A. Stable chromatin binding prevents FoxA acetylation, preserving FoxA chromatin remodeling. J. Biol. Chem. 2010; 285:464–472. 19897491 10.1074/jbc.M109.063149 PMC2804194

[45] Kardassis D., Falvey E., Tsantili P., Hadzopoulou-Cladaras M., Zannis V. Direct physical interactions between HNF-4 and Sp1 mediate synergistic transactivation of the apolipoprotein CIII promoter. Biochemistry (Mosc.). 2002; 41:1217–1228. 10.1021/bi015618f 11802721

[46] Xu L., Ma X., Bagattin A., Mueller E. The transcriptional coactivator PGC1α protects against hyperthermic stress via cooperation with the heat shock factor HSF1. Cell Death Dis. 2016; 7:e2102. 26890141 10.1038/cddis.2016.22 PMC5399192

[47] Hurgin V., Novick D., Rubinstein M. The promoter of IL-18 binding protein: activation by an IFN-gamma -induced complex of IFN regulatory factor 1 and CCAAT/enhancer binding protein beta. Proc. Natl. Acad. Sci. U.S.A. 2002; 99:16957–16962. 12482935 10.1073/pnas.262663399 PMC139251

[48] Kuwata T., Gongora C., Kanno Y., Sakaguchi K., Tamura T., Kanno T., Basrur V., Martinez R., Appella E., Golub T. Gamma interferon triggers interaction between ICSBP (IRF-8) and TEL, recruiting the histone deacetylase HDAC3 to the interferon-responsive element. Mol. Cell. Biol. 2002; 22:7439–7448. 12370291 10.1128/MCB.22.21.7439-7448.2002 PMC135656

[49] Leng R.-X., Wang W., Cen H., Zhou M., Feng C.-C., Zhu Y., Yang X.-K., Yang M., Zhai Y., Li B.-Z. Gene-gene and gene-sex epistatic interactions of MiR146a, IRF5, IKZF1, ETS1 and IL21 in systemic lupus erythematosus. PLoS One. 2012; 7:e51090. 23236436 10.1371/journal.pone.0051090 PMC3517573

[50] Drew P.D., Franzoso G., Becker K.G., Bours V., Carlson L.M., Siebenlist U., Ozato K. NF kappa B and interferon regulatory factor 1 physically interact and synergistically induce major histocompatibility class I gene expression. J. Interferon Cytokine Res. Off. J. Int. Soc. Interferon Cytokine Res. 1995; 15:1037–1045. 10.1089/jir.1995.15.1037 8746784

[51] Ziegler-Heitbrock L., Lötzerich M., Schaefer A., Werner T., Frankenberger M., Benkhart E. IFN-alpha induces the human IL-10 gene by recruiting both IFN regulatory factor 1 and Stat3. J. Immunol. Baltim. Md 1950. 2003; 171:285–290. 10.4049/jimmunol.171.1.285 12817009

[52] Dornan D., Eckert M., Wallace M., Shimizu H., Ramsay E., Hupp T.R., Ball K.L. Interferon regulatory factor 1 binding to p300 stimulates DNA-dependent acetylation of p53. Mol. Cell. Biol. 2004; 24:10083–10098. 15509808 10.1128/MCB.24.22.10083-10098.2004 PMC525491

[53] Roopra A., Sharling L., Wood I.C., Briggs T., Bachfischer U., Paquette A.J., Buckley N.J. Transcriptional repression by neuron-restrictive silencer factor is mediated via the Sin3-histone deacetylase complex. Mol. Cell. Biol. 2000; 20:2147–2157. 10688661 10.1128/mcb.20.6.2147-2157.2000 PMC110831

[54] Gutierrez S., Javed A., Tennant D.K., van Rees M., Montecino M., Stein G.S., Stein J.L., Lian J.B. CCAAT/enhancer-binding proteins (C/EBP) beta and delta activate osteocalcin gene transcription and synergize with Runx2 at the C/EBP element to regulate bone-specific expression. J. Biol. Chem. 2002; 277:1316–1323. 11668178 10.1074/jbc.M106611200

[55] Ciavatta D.J., Yang J., Preston G.A., Badhwar A.K., Xiao H., Hewins P., Nester C.M., Pendergraft W.F., Magnuson T.R., Jennette J.C. Epigenetic basis for aberrant upregulation of autoantigen genes in humans with ANCA vasculitis. J. Clin. Invest. 2010; 120:3209–3219. 20714105 10.1172/JCI40034 PMC2929711

[56] Kitabayashi I., Yokoyama A., Shimizu K., Ohki M. Interaction and functional cooperation of the leukemia-associated factors AML1 and p300 in myeloid cell differentiation. EMBO J. 1998; 17:2994–3004. 9606182 10.1093/emboj/17.11.2994 PMC1170639

[57] Chiang B.-T., Liu Y.-W., Chen B.-K., Wang J.-M., Chang W.-C. Direct interaction of C/EBPdelta and Sp1 at the GC-enriched promoter region synergizes the IL-10 gene transcription in mouse macrophage. J. Biomed. Sci. 2006; 13:621–635. 16871431 10.1007/s11373-006-9101-y

[58] Höcker M., Raychowdhury R., Plath T., Wu H., O'Connor D.T., Wiedenmann B., Rosewicz S., Wang T.C. Sp1 and CREB mediate gastrin-dependent regulation of chromogranin A promoter activity in gastric carcinoma cells. J. Biol. Chem. 1998; 273:34000–34007. 9852054 10.1074/jbc.273.51.34000

[59] Syddall C.M., Reynard L.N., Young D.A., Loughlin J. The identification of trans-acting factors that regulate the expression of GDF5 via the osteoarthritis susceptibility SNP rs143383. PLoS Genet. 2013; 9:e1003557. 23825960 10.1371/journal.pgen.1003557 PMC3694828

[60] Karlseder J., Rotheneder H., Wintersberger E. Interaction of Sp1 with the growth- and cell cycle-regulated transcription factor E2F. Mol. Cell. Biol. 1996; 16:1659–1667. 8657141 10.1128/mcb.16.4.1659 PMC231152

[61] Corominas R., Yang X., Lin G.N., Kang S., Shen Y., Ghamsari L., Broly M., Rodriguez M., Tam S., Trigg S.A. Protein interaction network of alternatively spliced isoforms from brain links genetic risk factors for autism. Nat. Commun. 2014; 5:3650. 24722188 10.1038/ncomms4650 PMC3996537

[62] Hu X., Li T., Zhang C., Liu Y., Xu M., Wang W., Jia Z., Ma K., Zhang Y., Zhou C. GATA4 regulates ANF expression synergistically with Sp1 in a cardiac hypertrophy model. J. Cell. Mol. Med. 2011; 15:1865–1877. 20874724 10.1111/j.1582-4934.2010.01182.x PMC3918043

[63] Xie R.-L., Gupta S., Miele A., Shiffman D., Stein J.L., Stein G.S., van Wijnen A.J. The tumor suppressor interferon regulatory factor 1 interferes with SP1 activation to repress the human CDK2 promoter. J. Biol. Chem. 2003; 278:26589–26596. 12732645 10.1074/jbc.M301491200

[64] Kyo S., Takakura M., Taira T., Kanaya T., Itoh H., Yutsudo M., Ariga H., Inoue M. Sp1 cooperates with c-Myc to activate transcription of the human telomerase reverse transcriptase gene (hTERT). Nucleic Acids Res. 2000; 28:669–677. 10637317 10.1093/nar/28.3.669 PMC102554

[65] Gartel A.L., Ye X., Goufman E., Shianov P., Hay N., Najmabadi F., Tyner A.L. Myc represses the p21(WAF1/CIP1) promoter and interacts with Sp1/Sp3. Proc. Natl. Acad. Sci. U.S.A. 2001; 98:4510–4515. 11274368 10.1073/pnas.081074898 PMC31865

[66] Natesampillai S., Fernandez-Zapico M.E., Urrutia R., Veldhuis J.D. A novel functional interaction between the Sp1-like protein KLF13 and SREBP-Sp1 activation complex underlies regulation of low density lipoprotein receptor promoter function. J. Biol. Chem. 2006; 281:3040–3047. 16303770 10.1074/jbc.M509417200

[67] Carver B.J., Plosa E.J., Stinnett A.M., Blackwell T.S., Prince L.S. Interactions between NF-κB and SP3 connect inflammatory signaling with reduced FGF-10 expression. J. Biol. Chem. 2013; 288:15318–15325. 23558680 10.1074/jbc.M112.447318 PMC3663551

[68] Roder K., Wolf S.S., Larkin K.J., Schweizer M. Interaction between the two ubiquitously expressed transcription factors NF-Y and Sp1. Gene. 1999; 234:61–69. 10393239 10.1016/s0378-1119(99)00180-8

[69] Smith K.T., Coffee B., Reines D. Occupancy and synergistic activation of the FMR1 promoter by Nrf-1 and Sp1 in vivo. Hum. Mol. Genet. 2004; 13:1611–1621. 15175277 10.1093/hmg/ddh172

[70] Formisano L., Guida N., Valsecchi V., Cantile M., Cuomo O., Vinciguerra A., Laudati G., Pignataro G., Sirabella R., Di Renzo G. Sp3/REST/HDAC1/HDAC2 complex represses and Sp1/HIF-1/p300 complex activates ncx1 gene transcription, in brain ischemia and in ischemic brain preconditioning, by epigenetic mechanism. J. Neurosci. Off. J. Soc. Neurosci. 2015; 35:7332–7348. 10.1523/JNEUROSCI.2174-14.2015 PMC6705442 25972164

[71] Ingram R.M., Valeaux S., Wilson N., Bouhlel M.A., Clarke D., Krüger I., Kulu D., Suske G., Philipsen S., Tagoh H. Differential regulation of sense and antisense promoter activity at the Csf1R locus in B cells by the transcription factor PAX5. Exp. Hematol. 2011; 39:730–740. 21549805 10.1016/j.exphem.2011.04.004

[72] Giannopoulou E.G., Elemento O. Inferring chromatin-bound protein complexes from genome-wide binding assays. Genome Res. 2013; 23:1295–1306. 23554462 10.1101/gr.149419.112 PMC3730103

[73] Chakravarty K., Wu S.-Y., Chiang C.-M., Samols D., Hanson R.W. SREBP-1c and Sp1 interact to regulate transcription of the gene for phosphoenolpyruvate carboxykinase (GTP) in the liver. J. Biol. Chem. 2004; 279:15385–15395. 14744869 10.1074/jbc.M309905200

[74] Lim K., Chang H.-I. O-GlcNAc inhibits interaction between Sp1 and sterol regulatory element binding protein 2. Biochem. Biophys. Res. Commun. 2010; 393:314–318. 20138838 10.1016/j.bbrc.2010.01.128

[75] Biesiada E., Hamamori Y., Kedes L., Sartorelli V. Myogenic basic helix-loop-helix proteins and Sp1 interact as components of a multiprotein transcriptional complex required for activity of the human cardiac alpha-actin promoter. Mol. Cell. Biol. 1999; 19:2577–2584. 10082523 10.1128/mcb.19.4.2577 PMC84050

[76] Look D.C., Pelletier M.R., Tidwell R.M., Roswit W.T., Holtzman M.J. Stat1 depends on transcriptional synergy with Sp1. J. Biol. Chem. 1995; 270:30264–30267. 8530443 10.1074/jbc.270.51.30264

[77] Rossi A., Mukerjee R., Ferrante P., Khalili K., Amini S., Sawaya B.E. Human immunodeficiency virus type 1 Tat prevents dephosphorylation of Sp1 by TCF-4 in astrocytes. J. Gen. Virol. 2006; 87:1613–1623. 16690926 10.1099/vir.0.81691-0

[78] Kim E., Yang Z., Liu N.-C., Chang C. Induction of apolipoprotein E expression by TR4 orphan nuclear receptor via 5΄ proximal promoter region. Biochem. Biophys. Res. Commun. 2005; 328:85–90. 15670754 10.1016/j.bbrc.2004.12.146

[79] Lee J.S., Galvin K.M., Shi Y. Evidence for physical interaction between the zinc-finger transcription factors YY1 and Sp1. Proc. Natl. Acad. Sci. U.S.A. 1993; 90:6145–6149. 8327494 10.1073/pnas.90.13.6145 PMC46884

[80] Lee D.-K., Suh D., Edenberg H.J., Hur M.-W. POZ domain transcription factor, FBI-1, represses transcription of ADH5/FDH by interacting with the zinc finger and interfering with DNA binding activity of Sp1. J. Biol. Chem. 2002; 277:26761–26768. 12004059 10.1074/jbc.M202078200

[81] Abramovitch S., Glaser T., Ouchi T., Werner H. BRCA1-Sp1 interactions in transcriptional regulation of the IGF-IR gene. FEBS Lett. 2003; 541:149–154. 12706836 10.1016/s0014-5793(03)00315-6

[82] Zhang Y., Dufau M.L. Repression of the luteinizing hormone receptor gene promoter by cross talk among EAR3/COUP-TFI, Sp1/Sp3, and TFIIB. Mol. Cell. Biol. 2003; 23:6958–6972. 12972613 10.1128/MCB.23.19.6958-6972.2003 PMC193922

[83] Vallian S., Chin K.V., Chang K.S. The promyelocytic leukemia protein interacts with Sp1 and inhibits its transactivation of the epidermal growth factor receptor promoter. Mol. Cell. Biol. 1998; 18:7147–7156. 9819401 10.1128/mcb.18.12.7147 PMC109296

[84] Plaisance V., Niederhauser G., Azzouz F., Lenain V., Haefliger J.-A., Waeber G., Abderrahmani A. The repressor element silencing transcription factor (REST)-mediated transcriptional repression requires the inhibition of Sp1. J. Biol. Chem. 2005; 280:401–407. 15528196 10.1074/jbc.M411825200

[85] Su D., Peng X., Zhu S., Huang Y., Dong Z., Zhang Y., Zhang J., Liang Q., Lu J., Huang B. Role of p38 MAPK pathway in BMP4-mediated Smad-dependent premature senescence in lung cancer cells. Biochem. J. 2011; 433:333–343. 21050181 10.1042/BJ20100404

[86] Kadam S., McAlpine G.S., Phelan M.L., Kingston R.E., Jones K.A., Emerson B.M. Functional selectivity of recombinant mammalian SWI/SNF subunits. Genes Dev. 2000; 14:2441–2451. 11018012 10.1101/gad.828000 PMC316972

[87] Liu W.-L., Coleman R.A., Ma E., Grob P., Yang J.L., Zhang Y., Dailey G., Nogales E., Tjian R. Structures of three distinct activator-TFIID complexes. Genes Dev. 2009; 23:1510–1521. 19571180 10.1101/gad.1790709 PMC2704470

[88] Gagliardi A., Mullin N.P., Ying Tan Z., Colby D., Kousa A.I., Halbritter F., Weiss J.T., Felker A., Bezstarosti K., Favaro R. A direct physical interaction between Nanog and Sox2 regulates embryonic stem cell self-renewal. EMBO J. 2013; 32:2231–2247. 23892456 10.1038/emboj.2013.161 PMC3746198

[89] Ambrosetti D.C., Basilico C., Dailey L. Synergistic activation of the fibroblast growth factor 4 enhancer by Sox2 and Oct-3 depends on protein-protein interactions facilitated by a specific spatial arrangement of factor binding sites. Mol. Cell. Biol. 1997; 17:6321–6329. 9343393 10.1128/mcb.17.11.6321 PMC232483

[90] Ishiguro A., Kassavetis G.A., Geiduschek E.P. Essential roles of Bdp1, a subunit of RNA polymerase III initiation factor TFIIIB, in transcription and tRNA processing. Mol. Cell. Biol. 2002; 22:3264–3275. 11971960 10.1128/MCB.22.10.3264-3275.2002 PMC133792

[91] Bockmühl Y., Patchev A.V., Madejska A., Hoffmann A., Sousa J.C., Sousa N., Holsboer F., Almeida O.F.X., Spengler D. Methylation at the CpG island shore region upregulates Nr3c1 promoter activity after early-life stress. Epigenetics. 2015; 10:247–257. 25793778 10.1080/15592294.2015.1017199 PMC4622987

[92] Chiang C.M., Roeder R.G. Cloning of an intrinsic human TFIID subunit that interacts with multiple transcriptional activators. Science. 1995; 267:531–536. 7824954 10.1126/science.7824954

[93] Zhou Z., Li X., Deng C., Ney P.A., Huang S., Bungert J. USF and NF-E2 cooperate to regulate the recruitment and activity of RNA polymerase II in the beta-globin gene locus. J. Biol. Chem. 2010; 285:15894–15905. 20236933 10.1074/jbc.M109.098376 PMC2871457

[94] Kiryu-Seo S., Kato R., Ogawa T., Nakagomi S., Nagata K., Kiyama H. Neuronal injury-inducible gene is synergistically regulated by ATF3, c-Jun, and STAT3 through the interaction with Sp1 in damaged neurons. J. Biol. Chem. 2008; 283:6988–6996. 18192274 10.1074/jbc.M707514200

[95] Noti J.D. Sp3 mediates transcriptional activation of the leukocyte integrin genes CD11C and CD11B and cooperates with c-Jun to activate CD11C. J. Biol. Chem. 1997; 272:24038–24045. 9295357 10.1074/jbc.272.38.24038

[96] Lim K., Chang H.-I. O-GlcNAc inhibits interaction between Sp1 and Elf-1 transcription factors. Biochem. Biophys. Res. Commun. 2009; 380:569–574. 19285002 10.1016/j.bbrc.2009.01.121

[97] Tsai E.Y., Falvo J.V., Tsytsykova A.V., Barczak A.K., Reimold A.M., Glimcher L.H., Fenton M.J., Gordon D.C., Dunn I.F., Goldfeld A.E. A lipopolysaccharide-specific enhancer complex involving Ets, Elk-1, Sp1, and CREB binding protein and p300 is recruited to the tumor necrosis factor alpha promoter in vivo. Mol. Cell. Biol. 2000; 20:6084–6094. 10913190 10.1128/mcb.20.16.6084-6094.2000 PMC86084

[98] Galvagni F., Orlandini M., Oliviero S. Role of the AP-1 transcription factor FOSL1 in endothelial cells adhesion and migration. Cell Adhes. Migr. 2013; 7:408–411. 10.4161/cam.25894 PMC3903684 24084233

[99] Rosmarin A.G., Luo M., Caprio D.G., Shang J., Simkevich C.P. Sp1 cooperates with the ets transcription factor, GABP, to activate the CD18 (beta2 leukocyte integrin) promoter. J. Biol. Chem. 1998; 273:13097–13103. 9582348 10.1074/jbc.273.21.13097

[100] Latinkić B.V., Zeremski M., Lau L.F. Elk-1 can recruit SRF to form a ternary complex upon the serum response element. Nucleic Acids Res. 1996; 24:1345–1351. 8614640 10.1093/nar/24.7.1345 PMC145793

[101] Liu X., Li H., Rajurkar M., Li Q., Cotton J.L., Ou J., Zhu L.J., Goel H.L., Mercurio A.M., Park J.-S. Tead and AP1 Coordinate Transcription and Motility. Cell Rep. 2016; 14:1169–1180. 26832411 10.1016/j.celrep.2015.12.104 PMC4749442

[102] Stewart M.D., Choi Y., Johnson G.A., Yu-Lee L., Bazer F.W., Spencer T.E. Roles of Stat1, Stat2, and interferon regulatory factor-9 (IRF-9) in interferon tau regulation of IRF-1. Biol. Reprod. 2002; 66:393–400. 11804954 10.1095/biolreprod66.2.393

[103] Wadman I.A., Osada H., Grütz G.G., Agulnick A.D., Westphal H., Forster A., Rabbitts T.H. The LIM-only protein Lmo2 is a bridging molecule assembling an erythroid, DNA-binding complex which includes the TAL1, E47, GATA-1 and Ldb1/NLI proteins. EMBO J. 1997; 16:3145–3157. 9214632 10.1093/emboj/16.11.3145 PMC1169933

[104] Huang S., Qiu Y., Stein R.W., Brandt S.J. p300 functions as a transcriptional coactivator for the TAL1/SCL oncoprotein. Oncogene. 1999; 18:4958–4967. 10490830 10.1038/sj.onc.1202889

[105] Honda K., Taniguchi T. IRFs: master regulators of signalling by Toll-like receptors and cytosolic pattern-recognition receptors. Nat. Rev. Immunol. 2006; 6:644–658. 16932750 10.1038/nri1900

[106] Ferreira R., Ohneda K., Yamamoto M., Philipsen S. GATA1 function, a paradigm for transcription factors in hematopoiesis. Mol. Cell. Biol. 2005; 25:1215–1227. 15684376 10.1128/MCB.25.4.1215-1227.2005 PMC548021

[107] Woon Kim Y., Kim S., Geun Kim C., Kim A. The distinctive roles of erythroid specific activator GATA-1 and NF-E2 in transcription of the human fetal γ-globin genes. Nucleic Acids Res. 2011; 39:6944–6955. 21609963 10.1093/nar/gkr253 PMC3167640

[108] Lee C.S., Friedman J.R., Fulmer J.T., Kaestner K.H. The initiation of liver development is dependent on Foxa transcription factors. Nature. 2005; 435:944–947. 15959514 10.1038/nature03649

[109] Bonzo J.A., Ferry C.H., Matsubara T., Kim J.-H., Gonzalez F.J. Suppression of hepatocyte proliferation by hepatocyte nuclear factor 4α in adult mice. J. Biol. Chem. 2012; 287:7345–7356. 22241473 10.1074/jbc.M111.334599 PMC3293558

[110] Rodda D.J., Chew J.-L., Lim L.-H., Loh Y.-H., Wang B., Ng H.-H., Robson P. Transcriptional regulation of nanog by OCT4 and SOX2. J. Biol. Chem. 2005; 280:24731–24737. 15860457 10.1074/jbc.M502573200

[111] Raich N., Clegg C.H., Grofti J., Roméo P.H., Stamatoyannopoulos G. GATA1 and YY1 are developmental repressors of the human epsilon-globin gene. EMBO J. 1995; 14:801–809. 7882983 10.1002/j.1460-2075.1995.tb07058.x PMC398146

[112] Yan C., Boyd D.D. Histone H3 Acetylation and H3 K4 Methylation Define Distinct Chromatin Regions Permissive for Transgene Expression. Mol. Cell. Biol. 2006; 26:6357–6371. 16914722 10.1128/MCB.00311-06 PMC1592829

[113] Rye M., Sætrom P., Håndstad T., Drabløs F. Clustered ChIP-Seq-defined transcription factor binding sites and histone modifications map distinct classes of regulatory elements. BMC Biol. 2011; 9:80. 22115494 10.1186/1741-7007-9-80 PMC3239327

[114] Calo E., Wysocka J. Modification of enhancer chromatin: what, how, and why. Mol. Cell. 2013; 49:825–837. 23473601 10.1016/j.molcel.2013.01.038 PMC3857148

[115] Kordula T., Rydel R.E., Brigham E.F., Horn F., Heinrich P.C., Travis J. Oncostatin M and the interleukin-6 and soluble interleukin-6 receptor complex regulate alpha1-antichymotrypsin expression in human cortical astrocytes. J. Biol. Chem. 1998; 273:4112–4118. 9461605 10.1074/jbc.273.7.4112

[116] Caminsky N.G., Mucaki E.J., Perri A.M., Lu R., Knoll J.H.M., Rogan P.K. Prioritizing variants in complete hereditary breast and ovarian cancer genes in patients lacking known BRCA mutations. Hum. Mutat. 2016; 37:640–652. 26898890 10.1002/humu.22972

[117] Mucaki E.J., Caminsky N.G., Perri A.M., Lu R., Laederach A., Halvorsen M., Knoll J.H.M., Rogan P.K. A unified analytic framework for prioritization of non-coding variants of uncertain significance in heritable breast and ovarian cancer. BMC Med. Genomics. 2016; 9:19. 27067391 10.1186/s12920-016-0178-5 PMC4828881

[118] Yardımcı G.G., Frank C.L., Crawford G.E., Ohler U. Explicit DNase sequence bias modeling enables high-resolution transcription factor footprint detection. Nucleic Acids Res. 2014; 42:11865–11878. 25294828 10.1093/nar/gku810 PMC4231734

[119] Machanick P., Bailey T.L. MEME-ChIP: motif analysis of large DNA datasets. Bioinformatics. 2011; 27:1696–1697. 21486936 10.1093/bioinformatics/btr189 PMC3106185

[120] Setty M., Leslie C.S. SeqGL Identifies Context-Dependent Binding Signals in Genome-Wide Regulatory Element Maps. PLoS Comput. Biol. 2015; 11:e1004271. 26016777 10.1371/journal.pcbi.1004271 PMC4446265

[121] Heinz S., Benner C., Spann N., Bertolino E., Lin Y.C., Laslo P., Cheng J.X., Murre C., Singh H., Glass C.K. Simple combinations of lineage-determining transcription factors prime cis-regulatory elements required for macrophage and B cell identities. Mol. Cell. 2010; 38:576–589. 20513432 10.1016/j.molcel.2010.05.004 PMC2898526

