# Supplementary material for: Discovery and validation of information theory-based transcription factor and cofactor binding site motifs
Source: Nucleic Acids Res. 2016 Nov 28;45(5):e27. doi: 10.1093/nar/gkw1036 (PMC5389469; doi:10.1093/nar/gkw1036)
Supplement: Supplementary Data [file gkw1036_supplementary_data.zip › nar-02060-met-n-2016-File008.docx]

**Discovery and Validation of Information theory-based Transcription Factor and Cofactor Binding Site Motifs**

**Supplementary Methods**

**Ruipeng Lu^1^, Eliseos J. Mucaki^2^ and Peter K. Rogan^1,2,3,4^**

**^1^ Department of Computer Science, ^2^ Department of Biochemistry, ^3^ Department of Oncology, Western University, London, Ontario, N6A 4L6, Canada, ^4^ Cytognomix Inc., London, Ontario, N5X 3X5, Canada**

1. **Bioinformatic tools**

**1.1) The Bipad algorithm**

**1.2) The Maskminent motif discovery pipeline**

**1.2.1) The Maskminent program versus the Bipad program**

**1.2.2) The flowchart of the pipeline and an example**

**1.2.3) The commands to run the Maskminent program and the Scan program**

**1.2.4) The mathematical formalization of the masking technique**

**1.2.5) The mathematical formalization of selecting top peaks**

**1.2.6) The distributions of** $\boldsymbol{R}_{\boldsymbol{i}}$ **values of binding sites in top peaks versus in bottom peaks**

**2) Statistical analyses on iPWMs**

**2.1) The derivation on the relationship between binding energy** $\left( \mathbf{log}_{\boldsymbol{2}} \boldsymbol{K}_{\boldsymbol{di}} \right)$ **and** $\boldsymbol{R}_{\boldsymbol{i}}$ **values**

**2.2) Evaluation of the linearity between binding energy and** $\boldsymbol{R}_{\boldsymbol{i}}$ **values using F tests**

**2.3) An example slope analysis**

**1) Bioinformatic tools**

**1.1) The Bipad algorithm**


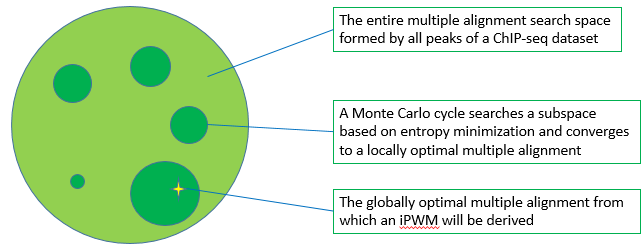


Figure 1. A high-level illustration of the Bipad algorithm

**Mathematical principles:**

Given the width $J$ of the iPWM to be derived, a peak dataset can be viewed as a multiple alignment search space $\theta$in which each multiple alignment whose size equals the number of peaks in the dataset is generated by taking a sequence fragment of length $J$ from each peak. Given a multiple alignment, the entropy of the position $l \left( 1\leq l\leq J \right)$ can be computed from:

$$E_{l}=\sum_{b\in B} f\left( b,l \right)\log_{2} \frac{1}{f\left( b,l \right)}, B=\left\{ A,C,G,T \right\} [1]$$

where $f\left( b,l \right)$ is the frequency of base $b$ at position $l$.

Bipad (1) uses a Monte Carlo framework to search subspaces in$\theta$, in an attempt to find the globally optimal multiple alignment with the minimum entropy (i.e. the maximum information content) in$\theta$. This process is independent of the nucleotide composition of input sequences. Therefore, the objective function is:

$$oMA=arg\min_{MA\in\theta} \left( E_{MA} \right) [2], E_{MA}=\sum_{l=1}^{J} E_{l} or\sum_{s\in\left\{ L,R \right\}} \left( \sum_{l=1}^{J_{s}} E_{l} \right) [3]$$

where $oMA$ is the optimal multiple alignment with the minimum entropy, $MA$ is one contiguous or bipartite multiple alignment in $\theta$, $E_{MA}$ is the entropy of $MA$, $J_{s}$ is the length of the left or right half site in $MA$.

**1.2) The Maskminent motif discovery pipeline**

**1.2.1) The Maskminent program versus the Bipad program**

The Maskminent program is obtained after adding the masking technique to the Bipad program. Therefore, it is a generalization of Bipad, and is able to reveal additional binding motifs that Bipad is not able to discover from the same ChIP-seq dataset.

**1.2.2) The flowchart of the pipeline and an example**


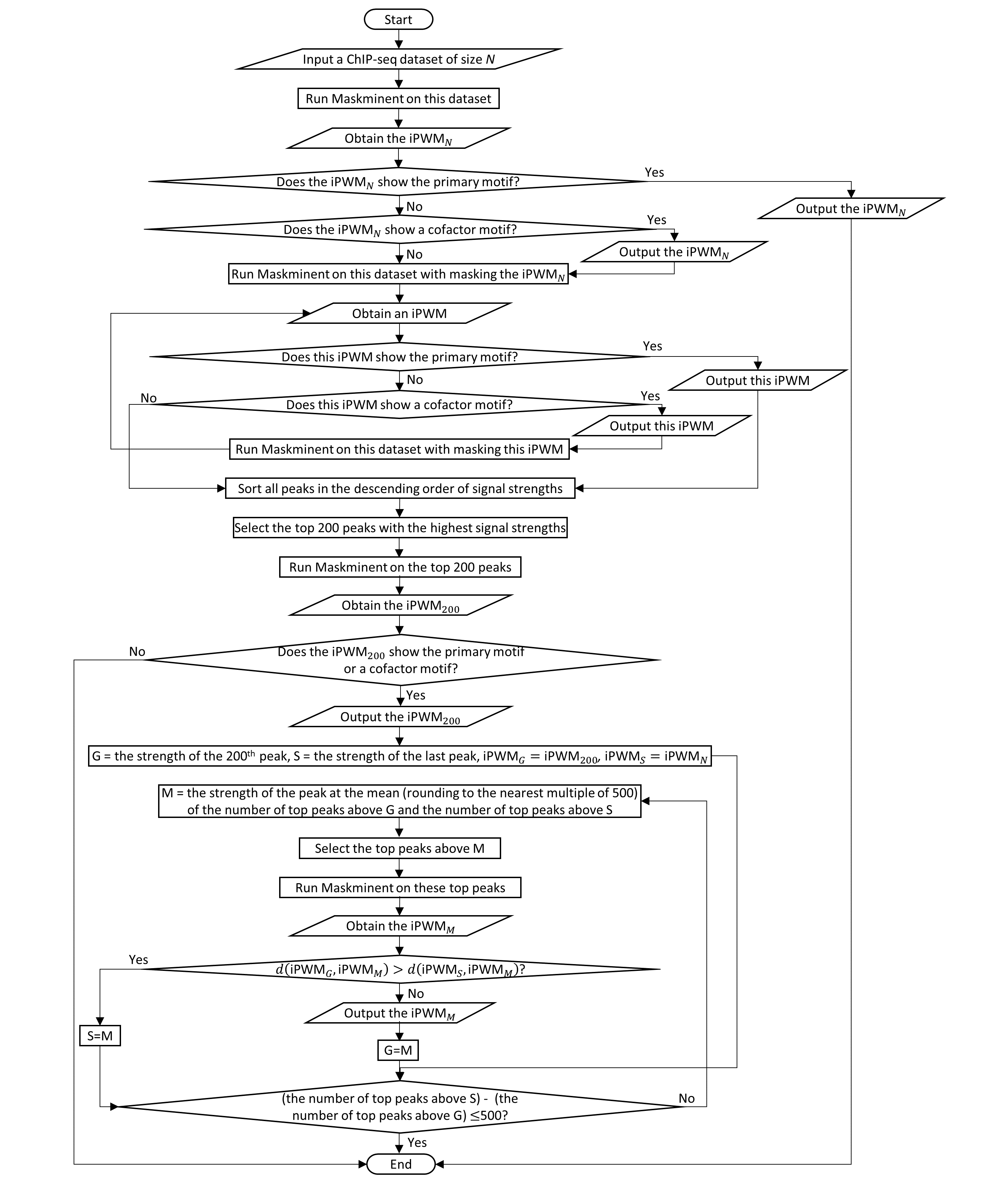


Figure 2. The flowchart of the Maskminent motif discovery pipeline *

* ${iPWM}_{N}$ is the initial iPWM derived from all peaks of the dataset. ${iPWM}_{200}$ is the iPWM derived from the top 200 peaks. The initial range containing the minimum threshold peak strength that can generate the primary/cofactor motif is from the strength of the 200^th^ peak (i.e. the initial value of $G$) to the strength of the last peak (i.e. the initial value of $S$). $S$ are the smaller bound of the current range containing the minimum threshold, and $G$ is the greater bound (i.e. the current threshold). ${iPWM}_{G}$, ${iPWM}_{S}$, ${iPWM}_{M}$ are respectively the iPWMs derived from the top peaks above $G$, $S$, $M$. $d\left( {iPWM}_{G},{iPWM}_{M} \right)$ is the Euclidean distance between ${iPWM}_{G}$ and ${iPWM}_{M}$, and $d\left( {iPWM}_{S},{iPWM}_{M} \right)$ is the Euclidean distance between ${iPWM}_{S}$ and ${iPWM}_{M}$. The approximately minimum threshold that we finally obtain is $G$ of the final range.

**Example**: Suppose we want to apply the Maskminent pipeline to a ChIP-seq dataset with 2,000 peaks. The following list shows each step of going through the above flowchart.

1. Derive ${iPWM}_{N}$ from all the 2,000 peaks.
2. ${iPWM}_{N}$ exhibits a noise motif.
3. Derive an iPWM ${iPWM}_{1}$ from all the 2,000 peaks with masking ${iPWM}_{N}$.
4. ${iPWM}_{1}$ exhibits a cofactor motif. So ${iPWM}_{1}$ is output.
5. Derive an iPWM ${iPWM}_{2}$ from all the 2,000 peaks with masking ${iPWM}_{N}$ and ${iPWM}_{1}$.
6. ${iPWM}_{2}$ exhibits a noise motif. Next we will use the thresholding technique.
7. Sort all the 2,000 peaks in the descending order of signal strengths.
8. Select the top 200 peaks.
9. Derive an iPWM ${iPWM}_{200}$ from the top 200 peaks.
10. ${iPWM}_{200}$ exhibits the primary motif. So ${iPWM}_{200}$ is output.
11. Assign the strength of the 200^th^ peak to$G$, and the strength of the 2,000^th^ peak to$S$. Assign ${iPWM}_{200}$ to ${iPWM}_{G}$, and ${iPWM}_{N}$ to ${iPWM}_{S}$.
12. Because 2,000-200>500, assign the strength of the 1,000^th^ peak to $M$. (1,000 = rounding (200+2,000)/2 to the nearest multiple of 500)
13. Select the top 1,000 peaks.
14. Derive ${iPWM}_{M}$ from the top 1,000 peaks.
15. $d\left( {iPWM}_{G},{iPWM}_{M} \right)$ is smaller than $d\left( {iPWM}_{S},{iPWM}_{M} \right)$. So ${iPWM}_{M}$ exhibits the primary motif, and it is output.
16. Assign $M$ (i.e. the strength of the 1,000^th^ peak) to $G$, and ${iPWM}_{M}$ to ${iPWM}_{G}$.
17. Because 2,000-1,000>500, assign the strength of the 1,500^th^ peak to $M$. (1,500 = rounding (1,000+2,000)/2 to the nearest multiple of 500)
18. Select the top 1,500 peaks.
19. Derive ${iPWM}_{M}$ from the top 1,500 peaks.
20. $d\left( {iPWM}_{G},{iPWM}_{M} \right)$ is smaller than $d\left( {iPWM}_{S},{iPWM}_{M} \right)$. So ${iPWM}_{M}$ exhibits the primary motif, and it is output.
21. Assign $M$ (i.e. the strength of the 1,500^th^ peak) to $G$, and ${iPWM}_{M}$ to ${iPWM}_{G}$..
22. Because 2,000-1,500=500, the pipeline is stopped.

Finally, the true TF binding motifs we obtain include a cofactor motif (i.e. ${iPWM}_{1}$) and the primary motif. The approximately minimum threshold that we finally obtain is the strength of the 1,500^th^ peak, and the most accurate iPWM exhibiting the primary motif that we obtain is derived on the top 1,500 peaks.

**1.2.3) The commands to run the Maskminent program and the Scan program**

In the Maskminent program, the –m option indicates whether to switch on masking, otherwise the commands specifying the run to build contiguous or bipartite iPWMs are similar to Bipad, respectively:

./Maskminent –n LengthOfiPWM –y NumberOfCycles –f ChIPseqFile [-m MaskFile] [-2]

./Maskminent –l LengthOfLeftHalfSite –r LengthOfRightHalfSite –a MinSpacerSize –b MaxSpacerSize -y NumberOfCycles –f ChIPseqFile –d/i

The command to run the Scan program is:

./Scan iPWMFile SequenceFile (1 LengthOfiPWM)/(2 LengthOfHalfSite MinSpacerSize MaxSpacerSize)

The ChIPseqFile consists of a set of DNA sequences, each of which may differ in length, as separate consecutive records.

**1.2.4) The mathematical formalization of the masking technique**

The masking technique is implemented by scanning a dataset with prior iPWMs, then recording the coordinates of all the predicted binding sites, and skipping these intervals when reanalyzing the dataset. This reduces the entire multiple alignment search space by the predicted sites of the preceding motifs. So the objective function of the Bipad algorithm (i.e. the Equation 2) changes accordingly:

$$oMA=arg\min_{MA\in\theta-\theta_{i}} \left( E_{MA} \right) [4]$$

where $\theta_{i}$ is the search space covered by the predicted binding sites of the previous iPWMs to be masked.

**1.2.5) The mathematical formalization of selecting top peaks**

Suppose that we want to select the top n peaks, after all peaks in a ChIP-seq dataset *D* are ranked in the descending order of signal strengths. *size(D)* is the number of peaks in *D*, and *min(D)* and *max(D)* are the lowest and highest signal values among all peaks in D, respectively. Then, the operation of thresholding the dataset *D* to obtain the top *n* peaks is defined by:

threshold(*D, n*) = $D_{n}, where \left( D_{n}\subset D \right)\wedge\left( size\left( D_{n} \right)=n \right)\wedge\left( min\left( D_{n} \right)\geq max\left( {D-D}_{n} \right) \right)$, *n < size(D)* [5]

The masking and thresholding techniques decrease the multiple alignment search space from distinct directions, as masking removing a portion in which the prior iPWMs are contained, and thresholding increasing the possibility of a non-noise motif possessing the smallest entropy in the remaining search space.

**1.2.6) The distributions of** $\boldsymbol{R}_{\boldsymbol{i}}$ **values of binding sites in top peaks versus in bottom peaks**

At the end of the thresholding process we obtain the approximately minimum threshold peak strength. The peak set above this threshold contains the maximum number of top peaks that can produce the primary/cofactor motif. However, the weak peaks below this threshold do not necessarily contain weak or missing binding sites. In fact, the distribution of *R_i_* values of binding sites in these bottom peaks is similar to that in the top peaks used to derive the primary motif. The fact that inclusion of the bottom peaks results in the disappearance of the primary motif implies that overall the binding sites in the top peaks have higher conservation levels (i.e. information contents). Below an example on the RUNX3 ChIP-seq dataset is given.

We took the greatest *R_i_* value in each peak after using the iPWM derived from the top 64,500 peaks to scan each peak in this dataset. The following graph shows the distributions of *R_i_* values for the top 64,500 peaks versus for the 3,465 bottom peaks.


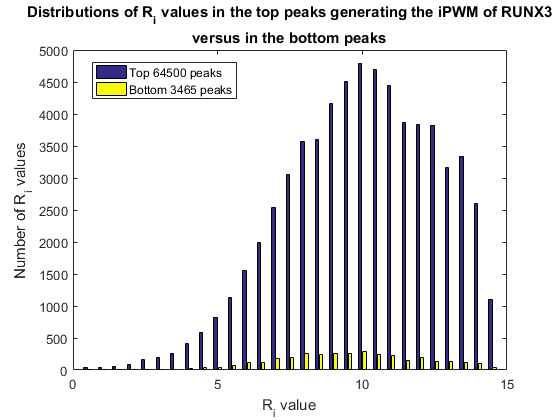


**2) Statistical analyses on iPWMs**

**2.1) The derivation on the relationship between binding energy** $\left( \mathbf{log}_{\boldsymbol{2}} \boldsymbol{K}_{\boldsymbol{di}} \right)$ **and** $\boldsymbol{R}_{\boldsymbol{i}}$ **values**

We found that the *R_i_* values are related to binding energy (or affinity). The frequencies of binding sites appearing in a ChIP-seq dataset are related to their binding energy (*log_2_K_d_*), which is delineated by Equation 6 derived by us starting from Levine et al. (2). And the *R_i_* values of binding sites are proportionate to their frequencies, since the stronger a binding site is, the more the number of times it is bound by the TF will be in a ChIP-seq assay.

$K_{di}\approx\frac{F_{1}^{'}K_{d1}}{F_{i}^{'}} [6]$

where $K_{di}$ is the dissociation constant of the $i$th predicted binding site, $F_{i}^{'}$ is the frequency of the $i$th site in a round of bounding (i.e. the frequency of the $i$th site appearing in the ChIP-seq dataset), $K_{d1}$ and $F_{1}^{'}$ are these values for the strongest site (i.e. the consensus sequence).

The Equation 6 was derived by recognizing that the thermodynamics of a population of TF-bound sequences is similar to a SELEX experimental framework (3). Below we give the detailed derivation.

From Levine et al. (2), first we obtain Equation 7:

$$\frac{F_{i}^{'}}{F_{1}^{'}}=\frac{K_{d1}+\left[ Tf \right]}{K_{di}+\left[ Tf \right]}\cdot\frac{F_{i}}{F_{1}} [7]$$

where $F_{i}$ is the frequency of the $i$th site in the prior round of bounding (i.e. in the genome), $F_{1}$ is this value for the strongest site (i.e. the consensus sequence), $\left[ Tf \right]$ is the concentration of unbound TF.

Multiply both sides of Equation 7 by $F_{1}^{'}$, then we obtain Equation 8:

$$F_{i}^{'}=\frac{K_{d1}+\left[ Tf \right]}{K_{di}+\left[ Tf \right]}\cdot\frac{F_{i}}{F_{1}}\cdot F_{1}^{'} [8]$$

Given that the consensus sequence is an extremely infrequent binding site both in the unselected population of binding sites and in the genome (3), we assume that its frequency will be similar during the early rounds of selection (i.e. $F_{1}=F_{1}^{'}$). Then, we obtain Equation 9:

$$F_{i}^{'}=\frac{K_{d1}+\left[ Tf \right]}{K_{di}+\left[ Tf \right]}\cdot F_{1}^{'} [9]$$

Solve for $K_{di}$, then we obtain Equation 10.

$$K_{di}= \frac{F_{1}^{'}\left( K_{d1}+[Tf] \right)}{F_{i}^{'}}-\left[ Tf \right] [10]$$

$\left[ Tf \right]$ is negligible for most TFs, because the concentrations of TFs inside cells are quite low (i.e. in the nM range) and $\left[ Tf \right]$ is only a fraction of them (4). Therefore, we obtain Equation 6 which suggests that the dissociation constants of binding sites are inversely proportional to their frequencies.

**2.2) Evaluation of the relationship between binding energy and** $\boldsymbol{R}_{\boldsymbol{i}}$ **values using F tests**

Higher $R_{i}$ values have lower binding energy. Primary/cofactor motifs are expected to demonstrate this relationship, whereas noise motifs are not. F tests can be used to evaluate this relationship, because they measure whether the linear regression model between the two variables provides a significantly better fit than the ‘nested’ constant regression model. A large F value means that the linear regression model has an slope well below 0, so primary/cofactor motifs are expected to have greater F values than noise motifs.

The frequency of a binding site appearing in the ChIP-seq dataset was calculated as follows: if only one binding site appeared in a peak, the frequency of this site would increase by one. In the case that a peak contained multiple binding sites, the frequency of each site would gain a fraction of one based on the proportion of its *R_i_* value accounting for the sum of *R_i_* values of all the sites.

For all the TFs we used 10^-7^M as the default value for the dissociation constant $K_{d1}$. This is because on an iPWM using different values for $K_{d1}$ will lead to the same F value. The detailed proof is given below.

Assume that for $K_{d1}$ we use two different values $K_{d1}^{a}$ and $K_{d1}^{b}$. According to Equation 6, we have

$$K_{di}^{a}\approx\frac{F_{1}^{'}K_{d1}^{a}}{F_{i}^{'}} \left[ 11 \right], K_{di}^{b}\approx\frac{F_{1}^{'}K_{d1}^{b}}{F_{i}^{'}} \left[ 12 \right]$$

Combining Equations 11 and 12, we obtain

$$K_{di}^{b}= \frac{K_{d1}^{b}}{K_{d1}^{a}}K_{di}^{a} [13]$$

Take the logarithm of both sides, then we obtain

$$\log_{2} K_{di}^{b}=\log_{2} K_{di}^{a}+\log_{2} \frac{K_{d1}^{b}}{K_{d1}^{a}} [14]$$

Equation 14 implies that in the graph of $R_{i}$ (X axis) versus binding energy (Y axis), the Y-axis values of all the data points will change the same amount ($\log_{2} \frac{K_{d1}^{b}}{K_{d1}^{a}}$) with the X-axis values remaining unchanged when $K_{d1}^{a}$ and $K_{d1}^{b}$ are used. This implies that the residual sum of squares (RSS) of the linear fitting model does not change when $K_{d1}^{a}$ and $K_{d1}^{b}$ are used, and the RSS of the constant fitting model does not change either. According to the following Equation 15 computing the F statistic, the resultant F-test value does not change when the two different values $K_{d1}^{a}$ and $K_{d1}^{b}$ are used.

$$F= \frac{\left( \frac{{RSS}_{1}-{RSS}_{2}}{p_{2}-p_{1}} \right)}{\left( \frac{{RSS}_{2}}{n-p_{2}} \right)} [15]$$

where ${RSS}_{1}$ is the RSS of the linear model, ${RSS}_{2}$ is the RSS of the constant model, $n$ is the number of data points (i.e. the number of binding sites in the iPWM), $p_{2}$ is the freedom of the linear model (i.e. 2), $p_{1}$ is the freedom of the constant model (i.e. 1).

Then we applied Equation 1 to compute the binding energy of each site.

**2.3) An example slope analysis**

For primary/cofactor motifs, the linear regression model between *R_i_* values and binding energy are expected to have slopes well below 0 which is the expected slope for noise motifs.

The following figure shows the probability density distributions of the slopes for 85 primary motifs, 85 cofactor motifs and 85 noise motifs that were randomly selected. The slopes of all primary motifs are smaller than -1.1 except that one is between -1 and -1.1, which also holds true for cofactor motifs; whereas the slopes of all cofactor motifs are greater than -1 except that one is between -1 and -1.1. This implies that the slope of the linear regression model between *log_2_K_d_* and *R_i_* can be used to distinguish primary/cofactor motifs from noise motifs.


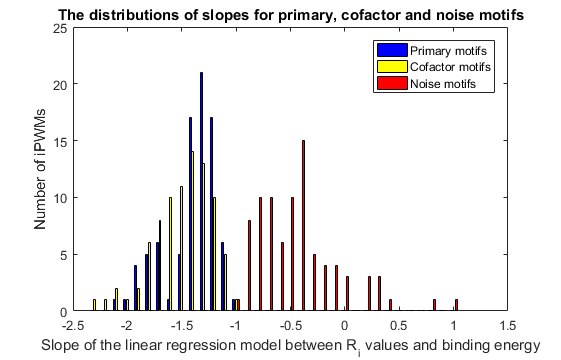


**References:**

1. Bi,C. and Rogan,P.K. (2004) Bipartite pattern discovery by entropy minimization-based multiple local alignment. *Nucleic Acids Res.*, **32**, 4979–4991.

2. Levine,H.A. and Nilsen-Hamilton,M. A mathematical analysis of SELEX. *Comput. Biol. Chem.*, **31**, 11–35.

3. Schneider,T.D. (1997) Information content of individual genetic sequences. *J. Theor. Biol.*, **189**, 427–441.

4. Sokolowski,T.R., Walczak,A.M., Bialek,W. and Tkačik,G. (2016) Extending the dynamic range of transcription factor action by translational regulation. *Phys. Rev. E*, **93**, 22404.
